# DNA Interactions and Biological Activity of 2,9-Disubstituted 1,10-Phenanthroline Thiosemicarbazone-Based Ligands and a 4-Phenylthiazole Derivative

**DOI:** 10.3390/biology13010060

**Published:** 2024-01-20

**Authors:** Álvaro Nicolás, Julia G. Quero, Marta Barroso, Zoila Gándara, Lourdes Gude

**Affiliations:** 1 Universidad de Alcalá, Departamento de Química Orgánica y Química Inorgánica, Instituto de Investigación Química “Andrés M. del Río” (IQAR), 28805 Madrid, Spain; a.nicolas@uah.es (Á.N.);; 2 Grupo DISCOBAC, Instituto de Investigación Sanitaria de Castilla-La Mancha (IDISCAM), 45071 Toledo, Spain

**Keywords:** thiosemicarbazones (TSCs), 1,10-phenanthroline, G4 DNA, antitumor agents, DNA interactions

## Abstract

**Simple Summary:**

This work describes the synthesis and characterization of four small-molecule derivatives based on the 1,10-phenanthroline heterocyclic system as potential telomeric quadruplex DNA ligands with antitumor properties. DNA-ligand interaction studies were carried out to determine thermal stabilization, structural effects and binding affinity towards the human telomeric DNA sequence Tel22. Cytotoxicity in tumor and normal cells was preliminarily tested, along with alterations in cell cycle and the ability to induce apoptosis. Three of the studied compounds can bind and stabilize the telomeric quadruplex sequence and exhibit moderate cytotoxicity in tumor cells. In addition, they were shown to induce apoptosis in HeLa cells, which highlights their potential applications as chemical tools for the development of novel antitumor agents.

**Abstract:**

Four 1,10-phenanthroline derivatives (**1**–**4**) were synthesized as potential telomeric DNA binders, three substituted in their chains with thiosemicarbazones (TSCs) and one 4-phenylthiazole derivative. The compounds were characterized using NMR, HRMS, FTIR-spectroscopy and combustion elemental analysis. Quadruplex and dsDNA interactions were preliminarily studied, especially for neutral derivative **1**, using FRET-based DNA melting assays, equilibrium dialysis (both competitive and non-competitive), circular dichroism and viscosity titrations. The TSC derivatives bind and stabilize the telomeric Tel22 quadruplex more efficiently than dsDNA, with an estimated 24-fold selectivity determined through equilibrium dialysis for compound **1**. In addition, cytotoxic activity against various tumor cells (PC-3, DU145, HeLa, MCF-7 and HT29) and two normal cell lines (HFF-1 and RWPE-1) was evaluated. Except for the 4-phenylthiazole derivative, which was inactive, the compounds showed moderate cytotoxic properties, with the salts displaying lower IC_50_ values (30–80 μM), compared to the neutral TSC, except in PC-3 cells (IC_50_ (**1**) = 18 μM). However, the neutral derivative was the only compound that exhibited a modest selectivity in the case of prostate cells (tumor PC-3 versus healthy RWPE-1). Cell cycle analysis and Annexin V/PI assays revealed that the compounds can produce cell death by apoptosis, an effect that has proven to be similar to that demonstrated by other known 1,10-phenanthroline G4 ligands endowed with antitumor properties, such as PhenDC3 and PhenQE8.

## 1. Introduction

Since the discovery of the double structure of DNA [1,2], numerous efforts have been made to understand the different biological functions and structures that nucleic acids can adopt. Currently, it is widely recognized that DNA is a structurally dynamic molecule, with the capacity to adopt a variety of alternative secondary structures apart from the double helix, such as DNA hairpins, Holliday junctions, cruciform, triplexes or G-quadruplexes (G4) [3], just to mention a few. Among them, DNA and RNA quadruplexes have been gaining prominence in recent years for their biologically relevant roles, including their establishment as therapeutic targets for cancer treatment [4], but also for many other applications in biomedicine and nanotechnology [5,6,7]. Regarding cancer, it is a leading cause of death worldwide, accounting for almost 10 million deaths in 2020, or nearly one in six deaths, with an estimated 19.3 million new cancer cases recorded that year [8].

The studies conducted by Bang and Gellert revealed that guanylic acid tends to aggregate in solution, resulting in jelly-like structures [9,10]. Subsequent studies showed that four guanylic acid molecules can be arranged to form a planar tetrad [11,12]. In this tetrad, each guanine can function as a donor and acceptor of two pairs of hydrogen bonds, called Hoogsteen H-bonds. In addition, these tetrads tend to stack on top of each other through π-π stacking interactions and are stabilized by positive physiological cations (mainly K^+^ and Na^+^), thus giving rise to three-dimensional non-canonical secondary structures designated as G-quadruplexes (G4s) [13]. G4s can be unimolecular or intermolecular and can adopt a wide variety of topologies arising from several combinations of strand direction and loop sequence composition [14]. G4s have been associated with many different cellular processes and are present in a vast number of relevant genomic regions: in telomeres, in replication origins, the 5′-UTR regions and in several oncogenic promoters. Among their biological applications, the consideration of telomeric quadruplexes as potential anticancer targets deserves special attention. Telomeres are structures found at the ends of chromosomes, made up of regions of non-coding DNA with highly guanine-rich repetitive sequences. Their main function is to conserve genetic information, preserving genome integrity and functionality over time. Specifically, telomeric DNA is composed of a repeat of nucleotides rich in guanines (TTAGGG for eukaryotic cells) and has a length of between 9000 and 15,000 nucleotides [15].

The activity of ribonucleoprotein telomerase is intimately ligated to the type of secondary DNA and RNA structures formed in the telomeric region. It is responsible for maintaining telomere length and DNA synthesis through the reverse transcription of an RNA template contained as part of its structure. In most somatic cells, there is a progressive shortening of their telomeres, while in embryonic, stem and epithelial cells, telomerase activity is upregulated. Interestingly, this enzyme is overexpressed in approximately 80–90% of human tumor cells. Therefore, inhibition of this enzyme has been postulated as a therapeutic strategy against cancer [16]. Several telomerase inhibitors have already entered clinical or preclinical trials [17,18].

The stabilization of telomeric G4 structures through small molecules can prevent telomerase function, triggering apoptosis in cancer cells. In general, these G4 ligands fulfil a series of structural characteristics, possessing (a) an aromatic π-system with enough surface to favor end-stacking interactions with the quadruplex, (b) an electronic deficiency near the center of the G-quadruplex, with a stabilizing effect similar to that produced by K^+^ or Na^+^ ions and (c) one or more flexible side-chains positively charged at physiological pH, providing additional interactions with the grooves and/or loops [19,20,21,22]. However, it should be noted that there are some examples in the literature, such as the case of the molecule funtumin which, without fulfilling the mentioned characteristics, can interact with G4s [23]. Considering these generally accepted structural features, a wide variety of molecules have been designed and studied as G4 ligands, mainly those belonging to three families: fused polyaromatic derivatives, macrocyclic compounds and non-fused aromatic systems. The most representative examples of compounds that have shown relevant antitumor properties [24,25,26] include 2,6-diamidoanthraquinone, BisA, BRACO-19 (trisubstituted acridine), PIPER (perylene diimide derivative), Telomestatin (macrocyclic compound), Pyridostatin, TmPyP4 (cationic porphyrin derivative), Quarfloxacin, CX-5461, 360A, PhenDC3, benzofuran derivatives, etc. From these, it is worth mentioning that Quarfloxacin and CX-5461 are currently in Phase I/II clinical trials [27,28].

**Figure 1:**
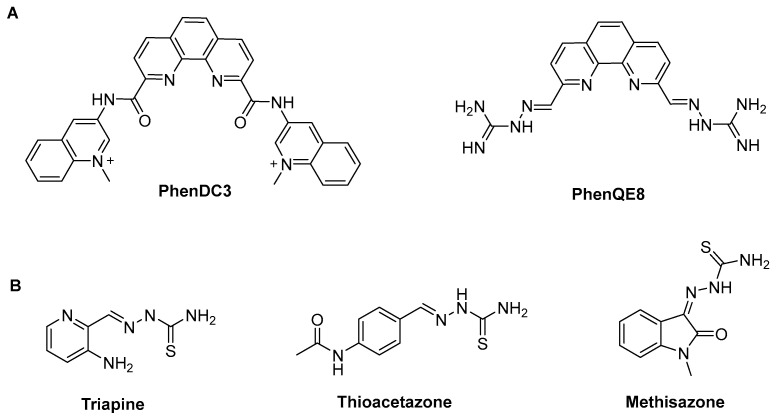
(**A**) Examples of 2,9-disubstituted 1,10-phenanthroline-based G4 ligands PhenDC3 and PhenQE8; (**B**) Examples of thiosemicarbazone (TSC-based) compounds with relevant biological activity [30,31].

We have recently reported a new G4 ligand derived from the 1,10-phenanthroline heterocycle (Figure 1A), compound PhenQE8 [29], which has proven cytotoxic in several types of tumor cells (PC-3, HeLa and MCF-7), with IC_50_ values in the low micromolar concentration range. At the same time, it exhibited lower cytotoxicity in the normal cells evaluated, HFF-1 and RWPE-1, demonstrating a good selectivity potential. As the interaction assays demonstrated its ability to selectively interact with telomeric DNA G-quadruplex compared to DNA duplex, our interest is now focused on the study of analogues of PhenQE8. We pursue, in this work, the synthesis and biological study of 1,10-phenanthroline derivatives substituted with hydrazinecarbothiamide (Thiosemicarbazone derivatives, TSC) or hydrazine 4-phenylthiazole chains.

It is important to highlight that thiosemicarbazones (TSCs) have been and continue to be used as versatile scaffolds for ligand synthesis in medicinal chemistry (Figure 1B). They have a wide range of biological activities including antibacterial, antifungal, antituberculosis, anticonvulsant, antioxidant and anticancer. In addition, they constitute the precursor structures for the synthesis of several types of heterocycles, such as 1,2,4-triazole-3-thiones, 4-thiazolidinones, oxadiazoles and 1,3,4-thiadiazoles, all of which are of enriched biological interest. Their mechanisms of action have been extensively studied but remain unclear. Some hypotheses that are worth mentioning include, among others, the inhibition of cellular iron uptake from Tf, iron mobilization from cells, the inhibition of ribonucleotide reductase activity, the up-regulation of the metastasis suppressor protein and the formation of redox active metal complexes that give rise to reactive oxygen species (ROS) [31,32]. Thiosemicarbazones have also been used in the form of bidentate ligands or Schiff bases with various metals, e.g., Pt(II) and Au(III), as antitumor agents, including G-quadruplex DNA or thioredoxin reductase as therapeutic targets [33].

**Figure 2:**
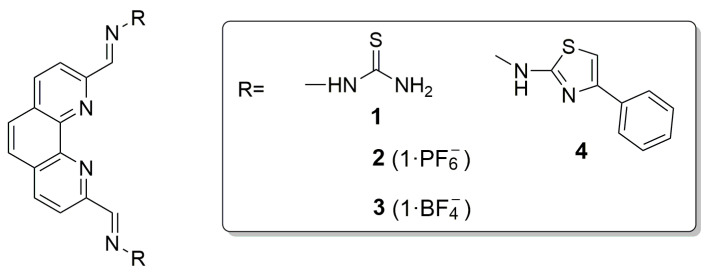
General structures of targeted thiosemicarbazones (TSCs) and 4-phenylthiazole-based analogue.

Based on the previous considerations, we report herein the preparation and characterization of three 2,9-disubstituted 1,10-phenanthroline compounds that contain hydrazinecarbothiamide in their side chains (thiosemicarbazones, TSCs), one as a neutral ligand and two as its corresponding PF_6_¯ and BF_4_¯ ionic salts (compounds **1**–**3**, Figure 2). Neutral ligand **1** is a known compound [34], and it was used as a precursor to obtain a heterocyclic derivative (compound **4**), in which the 1,10-phenanthroline is substituted with two hydrazine 4-phenylthiazole motifs. Quadruplex and dsDNA interactions were preliminarily studied, especially for neutral derivative **1**, using DNA FRET-based melting assays, equilibrium dialysis, circular dichroism (CD) and viscosity assays. In addition, cytotoxic activity against several tumor cells (PC-3, DU145, HeLa, MCF-7 and HT29) and two non-tumor cell lines (HFF-1 and RWPE-1) was tested. Cell cycle analysis and Annexin V/PI assays were carried out to preliminarily assess the effects on cell cycle phases and whether the novel compounds can produce cell death by apoptosis. Their biological activity was compared to other known 1,10-phenanthroline G4 ligands endowed with antitumor properties, such as PhenDC3 [35] and PhenQE8.

## 2. Materials and Methods

### 2.1. General Methods

All reagents and solvents were acquired from commercial sources in high purity form Merck (Madrid, Spain) BLD Pharm (Reinbek, Germany), Carlo Erba Reagents (Sabadell, Spain), PanReac AppliChem-ITW Reagents (Barcelona, Spain), Fisher (Alcobendas, Spain) Scharlab (Barcelona, Spain), Acros Organics (Alcobendas, Spain) and Thermo Scientific Chemicals (Alcobendas, Spain) and used as received. 1,10-phenanthroline-2,9-carbaldehyde was synthesized following a reported procedure [29]. Analytical TLC was performed on precoated aluminum silica gel sheets (Merck, Madrid, Spain, 60 F_254_ 0.25 mm). Melting points were measured on an Electrothermal Digital IA9100 equipment (VWR, Llinars del Vallès, Spain) and are reported uncorrected. IR spectra from powdered solids were recorded on an IR Bruker Alpha II spectrophotometer (Agilent Technologies, Madrid, Spain) under ambient conditions between 4000 and 400 cm^−1^ on a diamond ATR unit. ^1^H, ^13^C, ^31^P and ^19^F NMR spectra were registered on a Bruker 400 Ultrashield (Rivas Vaciamadrid, Spain) in DMSO-d_6_ at 298 K. Chemical shifts *(δ*) are reported considering DMSO-d_6_ (*δ*_H_ 2.50 ppm) and DMSO-d_6_ (*δ*_C_ 39.5 ppm). Coupling constant values (*J*) are reported in Hz. Signal multiplicity is designated with the notations s (singlet), bs (broad singlet), d (doublet), t (triplet), at (apparent triplet) or hept (heptuplet). Electron spray ionization–mass spectra (ESI-MS) were recorded on a MAXISII instrument (Bruker, Vaciamadrid, Spain, SiDI, Universidad Autónoma de Madrid, UAM). Combustion elemental analysis was conducted on a LECO CHNS-932 apparatus (Tres Cantos, Spain).

Short oligonucleotides sequences were acquired in high purity from IDT^®^ (Integrated DNA Technologies, Leuven, Belgium) and molecular biology grade reagents from Sigma-Aldrich (Merck, Madrid, Spain). DNA stock solutions were prepared using BPC-grade water. In all DNA interaction studies, Milli-Q water was employed for the preparation of the working buffers. Stock solutions of compounds **1**–**4** at ~1 mM concentration were made freshly immediately before each assay in water and a maximum 10% DMSO, and they were gradually diluted to the desired final concentrations in the corresponding buffer or cell culture medium.

UV-visible experiments were conducted using a Perkin-Elmer Lambda 35 spectrophotometer (Tres Cantos, Spain). CD spectra were recorded employing a Jasco J-715 spectropolarimeter (Madrid, Spain). FRET DNA melting assays were performed in microplate-format on a QuantStudio 6 PRO 0.2 mL, 96-well package (Applied Biosystems, Madrid, Spain). Flow times for viscosity determinations were recorded at 25 °C using a Visco System AVS 470 (Net Interlab, Madrid, Spain) with a microUbbelohde (K = 0.01018) capillary viscometer. MTT viability assays were performed by specialized personnel at the Cell Culture Unit (UAH) following standard reported protocols and using an ELISA plate reader (ELX 800 Biotech Instruments, Agilent Technologies, Madrid, Spain). Cell cycle and apoptosis assays were performed using flow cytometry on a MACSQuant^®^ Analyzer 10 Flow Cytometer (Miltenyi Biotech, Pozuelo de Alarcón, Spain).

### 2.2. Synthesis and Characterization of Chemical Compounds

#### 2.2.1. 2,2′-(1,10-Phenanthroline-2,9-diyl)-bis(methylene)-bis(hydrazine-1-carbothioamide) (**1**)

1,10-phenanthroline-2,9-carbaldehyde (98 mg, 0.42 mmol) was dissolved in dry MeOH (8 mL) with stirring, and then thiosemicarbazide (76 mg, 0.84 mmol) and 3 drops of 96% H_2_SO_4_ were added. The reaction mixture was then heated at reflux for 1 h, cooled at room temperature and filtered. The resulting solid was washed once with cold water, then with MeOH and finally three times with Et_2_O, and it was dried under vacuum. The solid was recrystallized using a mixture of 9:1 AcCN/MeOH, providing compound 1 (128 mg, 81%) as an orange solid. M.p.: >180 °C (decompn.). IR: v (cm^−1^): 3250, 3143, 2944, 1592, 1513, 1491, 1200, 1115, 1043. ^1^H-NMR (400 MHz, DMSO-d_6_): δ (ppm) 11.99 (s, 2H, NH), 8.77 (d, *J* = 8.6 Hz, 2H, H-4,7), 8.57 (d, *J* = 8.6 Hz, 2H, H-3,8), 8.51 (bs, 2H, NH), 8.48 (s, 2H, N=CH), 8.42 (bs, 2H, NH), 8.05 (s, 2H, H-5,6). ^13^C-NMR (101 MHz, DMSO-d_6_): δ (ppm) 179.1 (-C=S), 153.7 (C-2,9), 143.1 (C-10a,10b), 141.4 (CH=N), 138.4 (CH, C-4,7), 129.4 (C-4a,6a), 127.6 (CH, C-5,6), 121.1 (CH, C-3,8). HRMS (ESI, *m/z*) for C_16_H_15_N_8_S_2_ [M + H]^+^: calculated: 383.0861; found: 383.0857. HRMS (ESI, *m/z*) for C_16_H_14_N_8_S_2_Na [M + Na]^+^: calculated: 405.0680; found: 405.0673. Anal. Calcd. (%) for C_16_H_14_N_8_S_2_·6H_2_O: C, 39.17; H, 5.34; N, 22.84. Found: C, 38.77; H, 4.82; N, 17.47. 1,10-phenanthroline derivatives are known to crystallize as hydrates and/or solvates.

#### 2.2.2. 2,2′-(1,10-Phenanthroline-2,9-diyl)-bis(methylene)-bis(hydrazine-1-carbothioamide) Hexafluorophosphate Salt (**2**)

1,10-phenanthroline-2,9-carbaldehyde (50 mg, 0.21 mmol) was dissolved in dry MeOH (4 mL) with stirring, and then thiosemicarbazide (39 mg, 0.42 mmol) and 3 drops of 96% H_2_SO_4_ were added. The reaction mixture was heated at reflux for 1 h, and then KPF_6_ (0.42 mmol, 78 mg) was added. The mixture was then stirred at reflux for another 30 min and, additionally, 30 min at room temperature, after which the solid obtained was filtered and washed first with cool MeOH once, and then three times with Et_2_O. The product was recrystallized using a mixture of 9:1 AcCN/MeOH and filtered again to provide compound 2 (51 mg, 46%) as an orange solid. M.p.: >240 °C (decompn.). IR: v (cm^−1^): 3346, 3249, 3154, 2944, 1592, 1517, 1200, 1053, 845, 751. ^1^H-NMR (400 MHz, DMSO-d_6_): δ (ppm) 12.02 (s, 2H, NH), 8.79 (d, *J* = 8.6 Hz, 2H, H-4,7), 8.60 (d, *J* = 8.6 Hz, 2H, H-3,8), 8.52 (bs, 2H, NH), 8.49 (s, 2H, N=CH), 8.42 (bs, 2H, NH), 8.07 (s, 2H, H-5,6). ^13^C-NMR (101 MHz, DMSO-d_6_): δ (ppm) 179.0 (-C=S), 154.0 (C-2,9), 144.5 (C-10a, 10b), 142.4 (CH=N), 137.6 (CH, C-4,7), 129.3 (C-4,6a), 127.5 (CH, C-5,6), 120.7 (CH, C-3,8). ^19^F-NMR (376 MHz, DMSO-d_6_): δ (ppm) −70.14 (d, *J* = 710.4 Hz). ^31^P-NMR (162 MHz, DMSO-d_6_): δ (ppm) −144.20 (hept, *J* = 710.4 Hz). HRMS (ESI, *m*/*z*) for C_16_H_14_N_8_S_2_Na [M + Na]^+^: calculated: 405.0680; found: 405.0666. HRMS (ESI, *m/z*) for PF_6_¯ [M]^−^: calculated: 144.9641; found: 144.9646. Anal. Calcd. (%) for C_16_H_15_N_8_S_2_PF_6_: C, 36.37; H, 2.86; N, 21.20. Found: C, 38.11; H, 3.98; N, 19.77.

#### 2.2.3. 2,2′-(1,10-Phenanthroline-2,9-diyl)-bis(methylene)-bis(hydrazine-1-carbothioamide) Tetrafluoroborate Salt (**3**)

An analogous procedure than the one used to obtain compound **2** was used for the preparation of compound **3**, using the same starting materials (1,10-phenanthroline-2,9-carbaldehyde (50 mg, 0.21 mmol) in dry MeOH (4 mL), thiosemicarbazide (39 mg, 0.42 mmol) and 3 drops of 96% H_2_SO_4_) heating at reflux for 1 h and then adding NaBF_4_ (46 mg, 0.42 mmol). Compound 3 was obtained as an orange solid (51 mg, 52%). M.p.: >240 °C (decompn.). IR: v (cm^−1^): 3349, 3249, 3151, 2953, 1592, 1521, 1446, 1205, 1050, 861. ^1^H-NMR (400 MHz, DMSO-d_6_): δ (ppm) 12.01 (s, 2H, NH), 8.78 (d, *J* = 8.6 Hz, 2H, H-4,7), 8.59 (d, *J* = 8.6 Hz, 2H, H-3,8), 8.52 (bs, 2H, NH), 8.49 (s, 2H, N=CH), 8.43 (bs, 2H, NH), 8.08 (s, 2H, H-5,6). ^13^C-NMR (101 MHz, DMSO-d_6_): δ (ppm) 179.0 (-C=S), 153.9 (C-2,9), 144.2 (C-10b, 10a), 142.2 (CH=N), 137.7 (CH, C-4,7), 129.3 (C-4, 6a), 127.5 (CH, C-5, 6), 120.7 (CH, C-3,8). ^19^F-NMR (376 MHz, DMSO-d_6_): δ (ppm) −148.22, −148.28. HRMS (ESI, *m/z*) for C_16_H_14_N_8_S_2_Na [M + Na]^+^: calculated: 405.0680; found: 405.0669. HRMS (ESI, *m/z*) for BF_4_¯ [M]^−^: calculated: 87.0029; found: 87.0070. Anal. Calcd. (%) for C_16_H_15_N_8_S_2_BF_4_·3H_2_O: C, 36.65; H, 4.04; N, 21.37. Found: C, 38.09; H, 3.80; N, 19.81.

#### 2.2.4. 2,9-bis((*E*)-(2-(4-Phenylthiazol-2-yl)hydrazineylidene)methyl)-1,10-phenanthroline (**4**)

To a solution of 2,2′-(1,10-phenanthroline-2,9-diyl)-bis(methylene)-bis(hydrazine-1-carbothioamide) (30 mg, 0.16 mmol) in EtOH (8 mL) 2-bromoacetophenone (31 mg, 0.16 mmol) was added, and the resulting mixture was stirred under reflux for 24 h. Afterwards, the crude was cooled at room temperature and centrifuged to 1500 rpm for 5 min. The supernatant was extracted, and the resulting precipitate was filtered, washed with Et_2_O and dried under vacuum, obtaining compound 4 (34 mg, 75%) as an orange solid. M.p.: >250 °C (decompn.). IR: v (cm^−1^): 3245, 2839, 1540, 1439, 1267, 1147, 1037. ^1^H-NMR (400 MHz, DMSO-d_6_): δ (ppm) 8.73 (d, *J* = 8.0 Hz, 2H, H-4,7-phenanthroline), 8.60 (s, 2H, H-5,6-phenanthroline), 8.38 (d, *J* = 8.0 Hz, 2H, H-3,8-phenanthroline), 8.15 (s, 2H, H-thiazole), 7.90 (d, *J* = 7.6 Hz, 4H, H-2,6-phenyl), 7.50 (s, 2H, HC=N), 7.44 (at, *J* = 7.8 Hz, 4H, H-3,5-phenyl), 7.34 (at, *J* = 7.3 Hz, 2H, H-4-phenyl). ^13^C-NMR (101 MHz, DMSO-d_6_): δ (ppm) 167.8 (S-C=N), 152.8 (C=N, thiazole), 151.2 (C-2,9-phenanthroline), 140.0 (C-4a,6a-phenanthroline), 138.5 (CH=N), 134.7 (C-4,7-phenanthroline), 129.2 (C-3,5-phenyl), 128.8 (C-4-phenyl), 128.3 (C-5,6-phenanthroline), 128.3 (C-10a,10b-phenanthroline), 127.5 (C-3,8-phenanthroline), 126.0 (C-2,6-phenyl), 105.8 (C-S, thiazole). HRMS (ESI, *m*/*z*) for C_32_H_23_N_8_S_2_ [M + H]^+^: calculated: 583.1487; found: 583.1472. HRMS (ESI, *m*/*z*) for C_32_H_22_N_8_S_2_Na [M + Na]^+^: calculated: 605.1306; found: 605.1293.

### 2.3. DNA Interaction Studies and Biological Activity

#### 2.3.1. Fret-Based DNA Melting Assays

FRET DNA melting assays were conducted following standard reported procedures [29]. Briefly, the employed oligonucleotides F21T (5′-FAM—(GGG TTA)_3_GGG—TAMRA-3′) and F10T (5′-FAM—TAT AGC TA TA/Sp18/TA GCT ATA—TAMRA-3′) were dissolved in buffer at 0.25 μM concentration and annealed by heating at 90 °C for 5 min, and then immediately cooling to 0 °C, in the case of F21T, or slowly to rt (F10T) during a period of 3 hours. The solutions were allowed to stand at 4 °C at least overnight.

Tested compounds **1**–**4** were dissolved in water with up to a maximum 10% DMSO (50 μM stock solution). Each stock solution was then gradually diluted to reach the final concentrations in the assay from 0 to 10 μM and a fixed 0.2 μM concentration of target sequences F21T or F10T. The buffer used for G4 sequence F21T was 10 mM KCl, 90 mM LiCl and 10 mM Li-cacodylate, pH 7.3. The buffer employed for F10T was 100 mM LiCl and 10 mM Li-cacodylate.

The melting process program consisted of an incubation step at 24 °C for 5 min and then a temperature ramp (1 °C/min rate) with fluorescence recordings at every degree up to 95 °C. Subsequently, the microplate with the samples (50 μL) was incubated for 5 min at 96 °C and then gradually cooled by using a temperature ramp of −1 °C/min, with fluorescence measurements at every degree down to 25 °C. The melting and refolding (or renaturing) curves were registered based on the change in emission of the FAM (6-carboxyfluorescein) fluorophore, excited at 492 nm and emitting at 516 nm. Melting temperature values (T_m_) were determined from replicate experiments using normalized curves and considering mid-transition T_1/2_ temperatures.

#### 2.3.2. Tel22 CD Titration Spectra

CD experiments were performed as previously reported [29]. Unlabeled Tel22 telomeric quadruplex sequence (5′-A(GGGTTA)_3_GGG-3′) at 4 μM concentration was folded in the same conditions as those employed for FRET-based melting experiments, using the buffering system as for G4 F21T. Tel22 and ligand **1** solutions at DNA/ligand ratios ranging from 1:1 to 1:5 were freshly prepared before the assay and allowed to stand for 2 h prior to measurements. The temperature used in the titrations was kept at 25 °C during the experiments, and CD spectra were registered using a 0.5 cm-path cell, 1 nm band width and 0.5 nm increments.

#### 2.3.3. Non-Competition and Competition Equilibrium Dialysis Experiments

Equilibrium dialysis experiments were conducted with unlabeled quadruplex DNA and/or with dsDNA sequences as previously reported [29]. G4 sequence Tel22 is 5′-A(GGGTTA)_3_GGG-3′, and ds17 is 5′-GGG TTA CTA CGA ACT GG-3′/5′-CCA GTT CGT AGT AAC CC-3′. The buffering system employed contained 10 mM potassium phosphate buffer and 100 mM of KCl (pH = 7.2). DNA solution concentrations were determined at 90 °C through UV-visible spectrophotometry using the λ_max_ values and extinction coefficients provided by IDT^®^. Extinction coefficients of TSC ligand **1** were calculated in water and in buffer in the absence and in the presence of 1% (*w/v*) triton X-100. Competition and non-competition dialysis experiments were carried out using the protocol described by Chaires [36], with minor modifications. Dialysis bags (Biotech Regenerated Cellulose (RC) membrane, part number 133192, Spectrum Laboratories, Inc., Fisher, Madrid, Spain) with a 0.2 mL volume were used, with G4 Tel22 or duplex ds17 DNA at a concentration of 75 μM, in monomeric units. The dialysis bags were placed in a beaker containing 200 mL of a solution of compound **1** (2 μM) in buffer. The beaker was sealed with Parafilm, and the solution was allowed to equilibrate at room temperature (22 °C) for 24 h with continuous stirring. Then, the contents of the dialysis bags were transferred into microcentrifuge tubes, and triton X-100 was added (1% (*w/v*) final concentration). After 2 h equilibration at room temperature, the total concentration of the ligand (C_t_) was determined using UV-visible spectroscopy. The concentration of free compound **1** (C_f_) was also determined spectrophotometrically from the dialysate solution. The concentration of the DNA-bound compound (C_b_) was then calculated using the equation C_b_ = C_t_ − C_f_, and K_app_ was determined.

#### 2.3.4. CT Viscosity Assay

The DNA viscosity titration was conducted as previously reported [29]. For that purpose, *Calf thymus* CT DNA was used. DNA and compound **1** solutions were freshly prepared in 10 mM NaH_2_PO_4_/Na_2_HPO_4_ buffer, pH = 7.2. CT DNA solutions (~0.35 mM, nt) were equilibrated for 20 min at 25 °C in a thermostated bath, and 20 flow times were then recorded. Several aliquots (ca. 25–60 μL each) of a solution of ligand **1** (~1.8 mM) were sequentially added. After a 20 min equilibration at 25 °C, flow times were registered. Finally, viscosities (μ) were calculated at each titration point, from the averaged flow times recorded and the viscometer constant. The viscosity graphs were obtained by plotting (μ/μ_0_)^1/3^ versus r, the molar ratio of bound ligand to DNA. The parameter μ_0_ represents the DNA solution viscosity in the absence of a compound.

#### 2.3.5. Cell Culture Conditions and MTT Viability Assay

A panel of five tumor cell lines and two normal cell lines was used to preliminarily test the cytotoxicity of compounds **1**–**4**, and the experiments were conducted as previously reported [29]. All human cell lines were acquired from American Type Culture Collection (ATCC): tumor PC-3 and DU145 (prostate), HeLa (cervix), MCF7 (breast) and HT29 (colorectal) and healthy HFF-1 (fibroblast) and RWPE-1 (prostate). The cell catalogue reference and culture media used in the experiments were CRL-1435^TM^, RPMI (PC-3), HTB-81™, RPMI (DU145), CCL-2^TM^, DMEM (HeLa), HTB-22^TM^, DMEM (MCF-7), HTB-38^TM^, modified McCoy’s 5a medium, Catalogue No. 30-2007 (HT29), SCRC-1041^TM^, DMEM (HFF-1) and CRL-11609^TM^, Keratinocyte-SFM (RWPE-1). Culture media were purchased from Merck: RPMI-1640, DMEM + 10% FBS (Fetal Bovine Serum) + 10% antibiotic (penicillin/streptomycin/amphotericin B). Media and supplements for RWPE-1 were from ThermoFisher (17005075, Madrid, Spain). MCF7 cells were additionally supplemented with insulin (0.001 mg/mL in 500 mL media). Cells were kept at 37 °C (5% CO_2_), and culture media were renewed three times per week. When the cells reached 80–100% confluence, they were trypsinized and centrifuged, and the concentration was adjusted to each experiment. Cells were seeded at a density of ~10,000 cells/well into 24-well plates. The cells were treated with ligands **1**–**4** or the cytotoxic agents PhenQE8, PhenDC3 or cisplatin at different concentrations (10 nM–100 μM). Compound solutions were freshly prepared using the appropriate culture medium. Triplicate experiments were run. For MTT viability assays, after 72 h incubation, 50 μL of MTT (5 mg/mL) was added, and an additional incubation of 4 h was allowed. The medium in each well was then replaced by DMSO, and the absorbance was measured at 570 nm using an ELISA plate reader. Absorbances were normalized using negative controls (untreated cells, in triplicate, with a maximum of 0.9% DMSO content). Dose–response curves were obtained by plotting the percentage of viable cells versus logarithmic compound concentration. IC_50_ values were determined using Kaleidagragh^TM^ (3.52) software by adjusting the data to Equation 1/(1 + 10^(m2*(log(m1) − x))); m1 = 0.000003; m2 = 1.

#### 2.3.6. Cell Cycle and Annexin-V Apoptosis Assays

For cell cycle experiments, exponentially growing HeLa cells in DMEM were seeded in 6-well plates at a density of 2.5 × 10^5^/well. The cells were treated with compounds **1**, **2** or reference compounds with known antitumor activity (PhenDC3, 360A or PhenQE8), under several conditions: 72 h at ½ IC_50_ concentrations, 24 or 48 h at 20 μM. Untreated cells were used as controls. After washing with PBS, cells were detached with 0.25% trypsin/0.2% EDTA and centrifuged at 1500 rpm for 5 min at rt. The resulting pellets were mixed with cold 70% ethanol (0.5 mL) and kept at 4 °C for 30 min. Ethanol was removed by centrifugation, and the pellets were washed with 2 mL PBS + 2% BSA and centrifuged. After discarding the supernatants, a propidium iodide solution (PI/RNAse, FxCycle^TM^, Invitrogen^TM^ ref. F10797, 0.5 mL, Thermo Fisher, Madrid, Spain) was added, mixed thoroughly and incubated for 15 min at room temperature prior to analysis by flow cytometry. A total of 10,000 events were registered for each sample. The obtained results were analyzed with the MacsQuantify 2.13.1 program (Miltenyi Biotech, Pozuelo de Alarcón, Spain) and represented as histograms.

For the Annexin V apoptosis experiments [37], approximately half of the cells used in combined experiments with cell cycle analysis were harvested via trypsinization, PBS washed and stained with the Annexin-V FITC & PI kit (Invitrogen^TM^ ref. V 13242, Thermo Fisher, Madrid, Spain) following a standard protocol [38]. A total of 10,000 events were recorded, and the results obtained by flow cytometry were represented as dot plots.

## 3. Results

### 3.1. Synthesis and Characterization of 1,10-Phenanthroline TSCs and Derived 4-Phenylthiazole Analogue

**Scheme 1:**
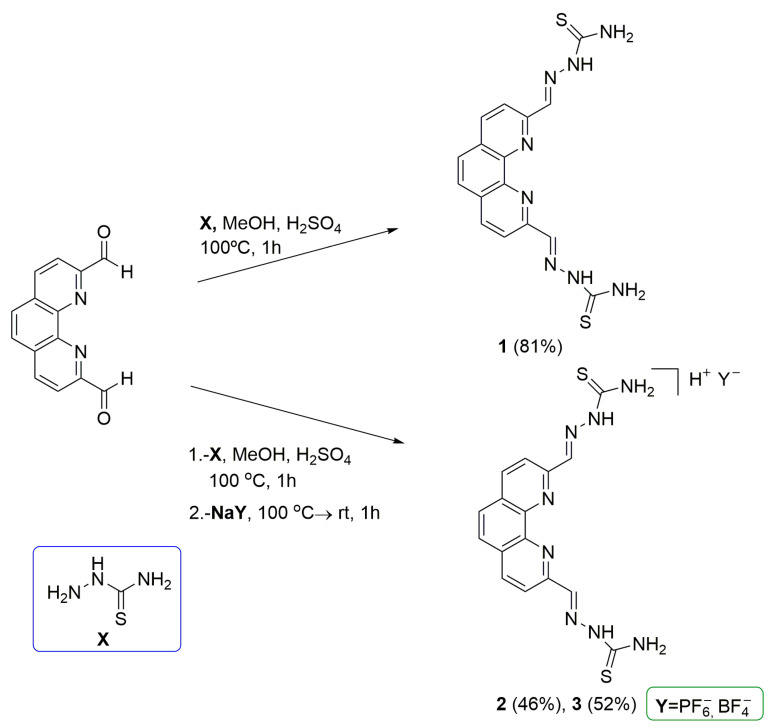
Synthesis of TSCs derivatives **1**–**3**.

The synthesis of 2,2′-((1,10-phenanthroline-2,9-diyl)bis(methanelylidene))-bis(hydrazine-1-carbothioamide) (**1**), and its corresponding PF_6_¯ and BF_4_¯ ionic salts, compounds **2** and **3**, respectively, was conducted via a straightforward and fast procedure from a precursor dialdehyde, 1,10-phenanthroline-2,9-dicarboxaldehyde. The dialdehyde was first prepared following established protocols, as previously reported [29]. Subsequently, through a condensation reaction with thiosemicarbazide (2 equiv., Scheme 1) in an acidic methanolic solution at reflux for 1h, in the absence of inorganic salts, compound **1** was obtained satisfactorily [34] in neutral form, with 81% yield.

**Scheme 2:**
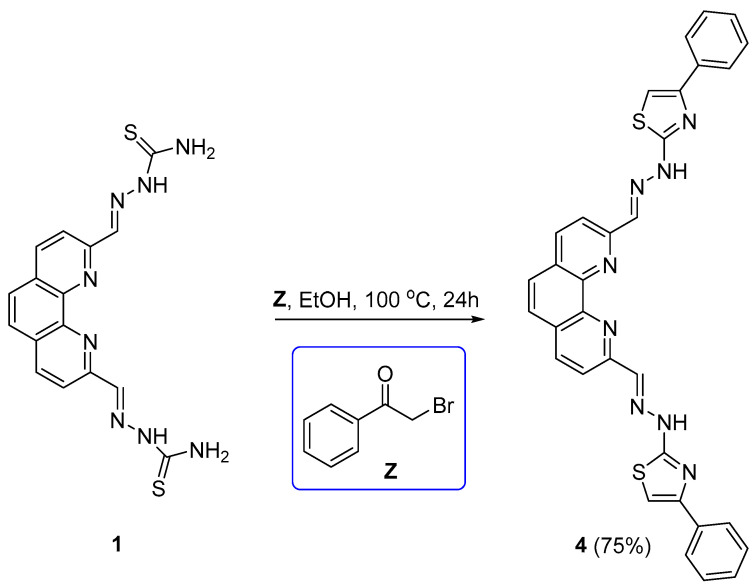
Synthesis of compound **4**.

In addition to the neutral compound **1**, two derived salts were prepared, the PF_6_¯ (**2**) and the BF_4_¯ (**3**) ionic salts. This was accomplished to study whether there is an effect on DNA interactions and/or biological activity, dependent on the existence and the nature of a counterion. As a matter of fact, it has been previously shown that altering the nature of the counterion in positively charged compounds can have significant effects, for instance, on cytotoxic activity [39,40,41]. Thus, the synthesis of TSC derivatives **2** and **3** was approached, and an analogous experimental procedure was followed by slightly varying the reaction conditions (Scheme 2). The process included an additional reaction step. After 1 h at reflux, a KPF_6_ or NaBF_4_ salt was added in stoichiometric excess (2 equiv.) to the reaction mixture and then cooled to room temperature, isolating the desired ionic derivative. Reaction yields were considerably lower for ionic TSCs, compared to the neutral compound: 46% for **2** (PF_6_¯ salt) and 52% for **3** (BF_4_¯ salt), respectively.

Finally, as thiosemicarbazones can be used as synthetic precursors to render heterocycles of biological relevance, compound **1** was used as the starting material for the synthesis of a thiazole derivative substituted with a benzene ring, compound **4** (2,9-bis((*E*)-(2-(4-phenylthiazol-2-yl)hydrazineylidene)methyl)-1,10-phenanthroline). In the absence of X-ray diffraction data, the isomer *E* is inferred through analogy to previous results with analogue PhenQE8. From a mechanistic perspective, the compound was obtained in good yield (75%) by the reaction of **1** with 2-bromoacetophenone in ethanol at reflux for 24 h (Scheme 2) through a Hantszch-type cyclodehydration reaction [42].

After purification by crystallization, the structural characterization of compounds **1**–**4** in solution was mainly conducted via ^1^H and ^13^C NMR using (CD_3_)_2_SO as a solvent (in the case of compounds **2** and **3**, additionally by ^31^P- and ^19^F-NMR), FTIR-spectroscopy, high-resolution MS spectrometry and combustion elemental analysis (Section 2 and Figures S1–S18).

### 3.2. DNA Interactions with Telomeric G4

#### 3.2.1. FRET-Based DNA Melting Experiments

Initial assessments of telomeric G4 thermal stabilization using compounds **1**–**4** was carried out through FRET-based DNA melting experiments. The sequence F21T [43], 5′-labeled with the fluorophore FAM and 3′-labeled with TAMRA, was utilized for that purpose, and the ds-DNA forming sequence F10T [44], labeled in a similar fashion, was used as a comparison for duplex stabilization. Both DNA sequences were titrated with compounds **1**–**4** at a concentration ranging from 1 μM to 10 μM.

**Figure 3:**
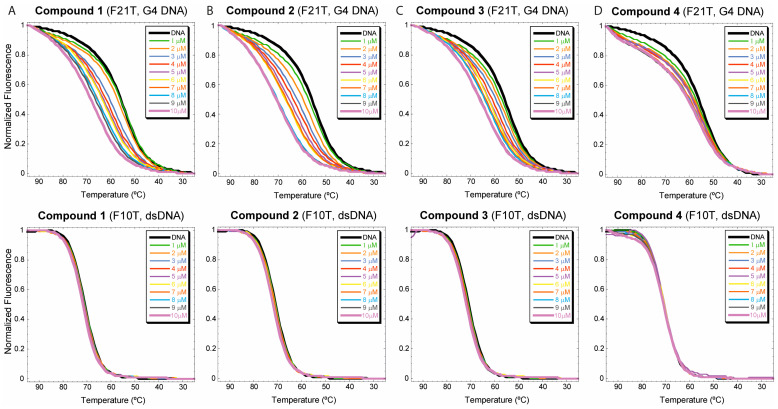
DNA FRET-based melting assays for derivatives **1**–**4** (panels (**A**–**D**), respectively). The telomeric sequence F21T (**top**) or dsDNA F10T (**bottom**) at 0.2 μM were treated with the tested compounds at concentrations between 1 and 10 μM. DNA renaturing curves are represented.

At the reference concentration of 1 μM, compounds **1**–**4** produced negligible stabilization effects. However, as the concentration of the compounds was augmented, all derivatives produced a dose-dependent increase in the T_m_ of the telomeric G4 sequence. Compounds **1**–**3** showed a similar behavior, with notable stabilization effects (ΔT_m_~7 °C, 9 °C and 6 °C at 5 μM; ΔT_m_~12 °C, 15 °C and 10 °C at 10 μM, respectively, Figure 3A–C, top panels), whereas compound **4** hardly stabilized the quadruplex structure (ΔT_m_~3 °C at 10 μM; Figure 3D, top panel). All the reported ΔT_m_ values refer to the effects produced by compounds **1**–**4** on the telomeric G-quadruplex renaturing process, starting from its denatured state to reach the folded conformation.

The thermal stabilizing effects produced by the TSC compounds were appreciable both in the DNA melting curves and in the opposite renaturing process, although the latter yielded better-quality sigmoidal curves. In general, these results highlight that compounds **1**–**3** are good stabilizing agents of the telomeric G-quadruplex structure, while derivative **4** produces only a subtle stabilization of the structure. In addition, when parallel experiments were run with dsDNA sequence F10T, none of the compounds showed stabilization of duplex structure (ΔT_m_~0 °C) up to the highest concentration tested, 10 μM (Figure 3A–D, bottom panels). These results support, for compounds **1**–**3**, the existence of a certain binding preference and higher stabilization effects towards the quadruplex structure than towards dsDNA. However, the establishment of binding selectivity would require additional experiments performed under competitive conditions.

#### 3.2.2. Circular Dichroism

Having detected that compounds **1**–**3**, the TSC compounds, can stabilize the telomeric quadruplex sequence, we were interested in assessing whether the core heterocyclic structure of these derivatives can induce structural changes on the quadruplex structure. Compound **1** was selected for further DNA-ligand interaction studies, starting with circular dichroism (CD) experiments employing the well-known telomeric sequence Tel22 (5′-A(GGGTTA)_3_GGG-3′). Although CD spectra do not provide precise information of G4 folded structures and their binding complexes, they can be utilized as a qualitative estimation of induced changes in the characteristic G4 folding topologies under a wide range of experimental conditions [45,46,47].

**Figure 4:**
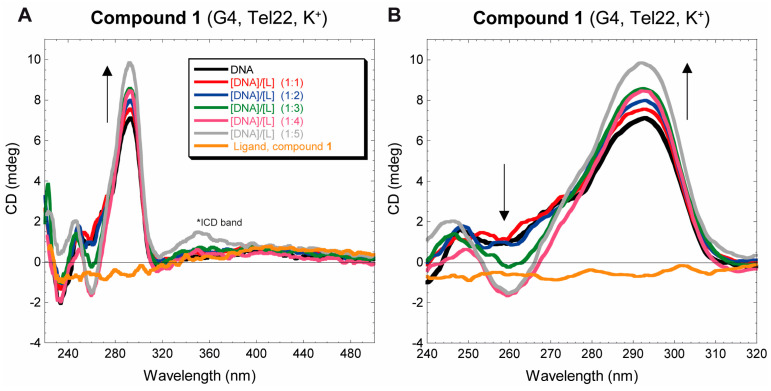
(**A**) Tel22 CD spectra (4 μM) folded under potassium ionic conditions after addition of compound **1** at molar ratios Tel22/ligand between 1:1 and 1:5. The presence of an ICD (induced circular dichroism) band at ~350 nm can be inferred at DNA/ligand ratio 1:5. (**B**) Zoomed view of CD spectrum (240–320 nm region).

The effects of compound **1** on the telomeric quadruplex structure were analyzed by recording the Tel22 CD spectra, folded under K^+^ ionic conditions, using various molar [DNA]/[ligand **1**] ratios, from 1:1 to 1:5. The main modifications of Tel22 CD signature are depicted in Figure 4. The intensity of the 295 nm band increased in a dose-dependent fashion, up to the maximum ligand/DNA ratio evaluated, molar ratio 1:5, whereas the intensity of the 270 nm band diminished. Overall, the spectral changes mentioned, including the appearance at ~260 nm of a negative signal, indicate that compound **1** stabilizes the telomeric G4 and, most importantly, the TSC derivative produces conformational changes in the G4 structure, increasing its antiparallel character. This observation is in good agreement with previously obtained results with compound PhenQE8 [29].

However, two main differences were detected in the CD experiments, when compared to compound PhenQE8. On the one hand, unlike PhenQE8, spectral changes associated with the stabilization of the structure with compound **1** were observed at DNA/ligand molar ratios higher than 2:1. On the other hand, an apparent ICD band (induced circular dichroism) was observed at 350 nm, at 1:5 molar ratio of DNA/ligand. This suggests that, besides the expected end-stacking binding mode of compound **1**, it is feasible that, when added in excess, the compound may bind the quadruplex structure in the grooves or undergo partial intercalation.

#### 3.2.3. Non-Competition and Competition Equilibrium Dialysis

We were then interested in establishing the apparent binding association constant of compound **1** and telomeric sequence Tel22 [48]. To this end, equilibrium dialysis experiments under non-competitive and competitive conditions were carried out. For comparison purposes and to estimate quadruplex/dsDNA selectivity, ds17 sequence [49] was also studied in both conditions. For the competitive assays, the experimental protocol developed by Chaires [36] was followed, with slight modifications (Section 2).

The results obtained with compound **1** with telomeric G4 Tel22 and duplex DNA ds17 are presented in Table 1. Assays were performed at room temperature with ~2 μM solutions of **1**, equilibrated with 75 μM (in monomeric units, tetrads or base pairs, respectively) of the tested DNA sequence for 24 h, in a buffer containing 100 mM KCl. Afterwards, the surfactanct triton X-100 was added to the solutions contained in the dialysis bags, and the recording of the UV-visible spectra was used to determine the concentrations of free and DNA-bound ligands for each situation: Tel22, ds17 or competition experiment Tel22-ds17. The concentration of DNA-bound ligand was determined based on at least two different experiments performed in duplicate or triplicate. The concentration data were then used to determine the apparent association constants of compound **1** with Tel22, with ds17 or with both structures simultaneously, in the competitive version using the equation K_app_ = C_b_/(C_f_)(S_total_ − C_b_) [36], where C_b_ is the concentration of ligand bound, C_f_ is the free ligand concentration and S_total_ = 75 μM (total DNA concentration), in monomeric units.

The determined K_app_ values are shown in Table 1. Compound **1** binds the telomeric sequence with an association constant in the order of 10^5^ M^−1^, and dsDNA ds17 with diminished affinity, in the order of 10^3^ M^−1^. In both competitive and non-competitive conditions, the compound demonstrated a higher binding affinity for the quadruplex sequence than for dsDNA sequence ds17, displaying a certain degree of selectivity. The determined apparent association constants under competitive conditions are in good agreement with those determined under non-competitive conditions. Concerning binding selectivity (determined by the quotient K_app_ (Tel22)/K_app_ (ds17) in the competitive assay), compound **1** exhibited preferential binding to the telomeric G4 over dsDNA (ca. 24-fold selectivity). This selectivity is considerably lower than the one previously reported for compound PhenQE8 (90-fold) [29]. Overall, the structural molecular difference that implies the substitution of two nitrogen atoms by two sulfur atoms, with changes in compound net charge, is reflected by a lower binding affinity to both G-quadruplex and dsDNA secondary structures.

#### 3.2.4. CT DNA Viscosity Titration

As the previous interaction assays revealed that the TSCs derivatives can recognize both quadruplex and dsDNA structures, we set out to study whether the binding mode towards double-stranded DNA occurs either by DNA intercalation, groove and/or electrostatic external binding or both. Viscosity titration assays are known to provide useful information, mainly in reversible dsDNA-ligand non-covalent binding modes [50], and, especially, they are normally used to differentiate between classical DNA intercalants from groove or external binders. The theory of Cohen and Eisenberg [51] establishes that the existence (or not) of changes in the viscosity of a DNA solution upon binding to small-molecule ligands could be correlated to the ligand binding mode. Therefore, DNA-ligand viscosity titrations can be employed to produce graphs representing the cubed root of the relative DNA viscosity (η/η_o_)^1/3^ against the molar ratio of the bound ligand to DNA (r). If a linear behavior is observed, the obtained slope value can be broadly associated with the DNA-ligand interaction mode. Groove and electrostatic external binders do not normally affect DNA viscosity and commonly display viscosity slope values of ~0.0. On the other hand, classical monointercalating agents, such as ethidium bromide, which increase the viscosity of the DNA solution upon binding, give rise to slope values of ~1.0 [50,51]. In practice, the slope values experimentally obtained for a great variety of known minor-groove binders oscillate from −0.3 to 0.2 [52].

**Figure 5:**
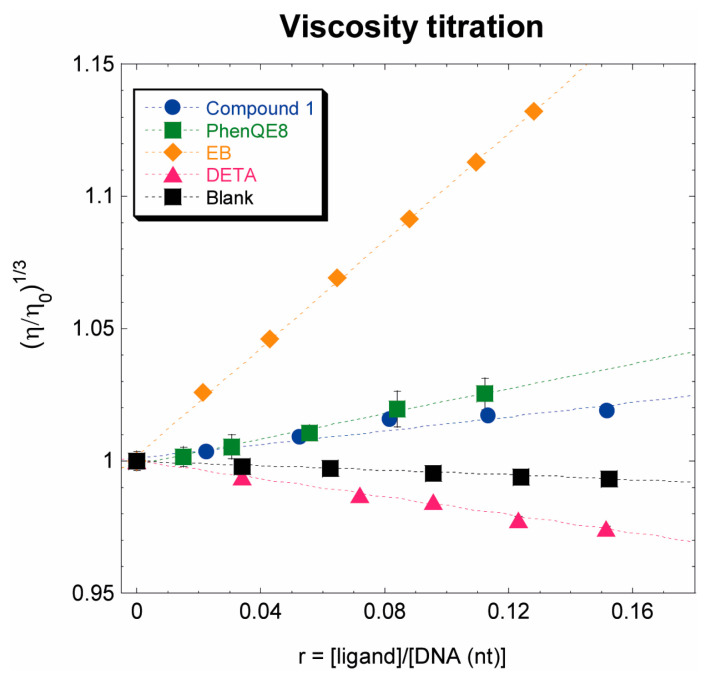
Calf Thymus (CT) DNA viscosity titration assay with ligand **1** at 25 °C. Reference compounds include EB: ethidium bromide and DETA: diethylenetriamine. PhenQE8 titrations are shown for comparison purposes. Blank represents the effects on DNA viscosity due to DNA dilution in titration experiments, using 10 mM sodium phosphate buffer (pH 7.2).

Compound **1** was chosen as the representative compound from the series, and viscometric titrations were performed in a thermostated bath (25 ± 0.01 °C) with solutions of Calf Thymus (CT) DNA and gradual additions of small aliquots of **1**. After an initial 20 min equilibration and further equilibrations following each subsequent addition, at least twenty flow times were registered in the absence (η_o_) or in the presence (η) of a ligand. Flow time results were converted to viscosity values, which were plotted as the relative viscosity values (η/η_o_)^1/3^ relative to r (ratio [ligand]/[DNA (nt)]), as displayed in Figure 5.

Compound **1** barely affected the CT DNA viscosity, showing a minor and linear effect on the [ligand]/[DNA] tested ratios, with a mean slope value of ca. 0.13, slightly lower than the one determined for compound PhenQE8 in previous studies (ca. 0.24). Similar to what was observed with PhenQE8 [29], and consistent with their similar structures, these results indicate that compound **1** does not bind CT dsDNA through the intercalation and insertion of the 1,10-phenanthroline ring between base pairs, but it interacts with dsDNA as a groove or as an electrostatic external binder.

### 3.3. Biological Activity: MTT, Cell Cycle and Apoptosis Assays

Finally, the cytotoxic activity and potential antitumor properties of compounds **1**–**4** in cultured cells were preliminarily evaluated through the MTT viability assay and, in the case of compounds **1**–**2**, through additional cell cycle and apoptosis assays. A panel of five human tumor cells was selected for this purpose: PC-3 and DU145 (prostate), HeLa (cervix), MCF-7 (breast) and HT29 (colorectal). As comparison and controls for cytotoxicity in non-tumor cells, two additional cell lines were also tested: fibroblasts (HFF-1) and prostate (RWPE-1). The cytotoxic activity of previously reported structural analogue PhenQE8 derivative, the highly selective G-quadruplex ligand and structurally related compound PhenDC3, and metallodrug cisplatin was also studied to preliminarily assess the biological activity of compounds **1**–**4**.

**Figure 6:**
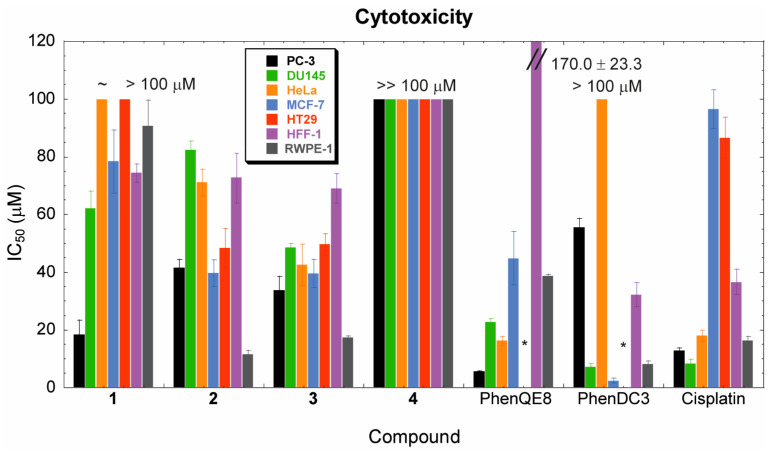
Bar graph representing IC_50_ values after 72 h, determined via the MTT assay. Cytotoxicity of compounds **1**–**4** and selected known antitumor compounds (PhenQE8, PhenDC3 and cisplatin) was tested in a panel of five tumor and two normal cell lines up to 100 μM concentration (except for PhenQE8, HFF-1 line). (*) Denotes not determined (HT29 cells).

The results are shown in Figure 6 and Table 2. With the exception of compound **4**, which proved to be non-cytotoxic in all cell lines tested, the rest of the compounds, after 72 h, exhibited moderate cytotoxicity at micromolar concentrations. Interestingly, neutral derivative **1** did not show cytotoxic activity in HeLa and in HT29 up to a 100 μM concentration, and, overall, its determined cytotoxicity in the cell lines evaluated, with the exception of PC-3, was lower than the one observed for compounds **2** and **3**, representing the same core structure but in the form of ionic salts. As a matter of fact, compounds **2** and **3** were found to be highly cytotoxic in normal RWPE-1 cells, especially compound **3**. Compound **1**, on the contrary, was the only derivative in the series with reduced cytotoxicity in a non-tumor cell line, RWPE-1. Overall, compounds **1**–**3** were less cytotoxic via the MTT assay than PhenQE8 and cisplatin in PC-3, DU145 and HeLa cells, but **2** and **3** showed similar or even improved activity in MCF7 and HT29 cells. When compared to PhenDC3, derivatives **1**–**3** were more cytotoxic in PC-3 cells and **2** and **3** in HeLa cells (Figure 6, Table 2). Finally, regarding the cytotoxicity in normal cells, derivatives **1**–**3** revealed lower cytotoxicity than PhenDC3 and cisplatin in the normal cells tested, especially in HFF-1 and, in particular, compound **1**. However, none of the derivatives demonstrated the selectivity previously reported for compound PhenQE8 [29].

Neutral derivative **1** and a representative example of an ionic salt, compound **2**, were selected for further preliminary studies of their potential antitumor properties. Thus, cell cycle experiments using flow cytometry were performed first. Tumor HeLa and PC3 cells were treated with compound **1**, compound **2** or selected antitumor controls (PhenQE8, 360A and/or PhenDC3) at ½ IC_50_ equivalent concentrations for 72 h, and changes in the cell cycle distribution (SubG0, G0/G1, S or G2/M populations) were quantitated. In HeLa cells, compounds **1** and **2** notably increased the number of cells in SubG0, which suggests DNA fragmentation and apoptosis, while they induced a decrease in the number of cells in the G0/G1 phase (Figure S19). Interestingly, very minor alterations in the cell cycle phase distribution were found for the tested compounds and controls in PC-3 cells under the experimental conditions previously used in the MTT viability assays.

**Figure 7:**
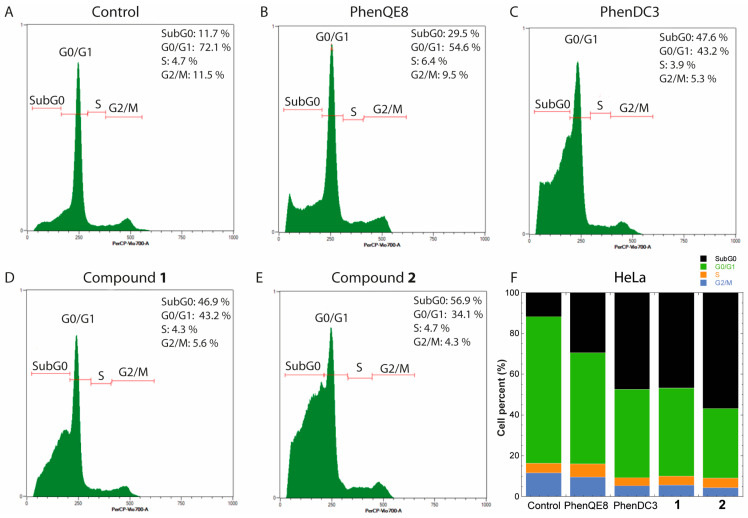
HeLa cell cycle histograms after 48 h treatment with 20 μM compound concentration. (**A**) Untreated cells (negative control); (**B**) PhenDC3 (antitumor agent, positive control); (**C**) PhenQE8 (structural analogue with antitumor properties, positive control); (**D**) compound **1**; (**E**) compound **2**; and (**F**) stack bar graph representing averaged cell percents in each cycle phase and SubG0 after compound treatment. Increase in the SubG0 population percent is associated with apoptosis.

**Figure 8:**
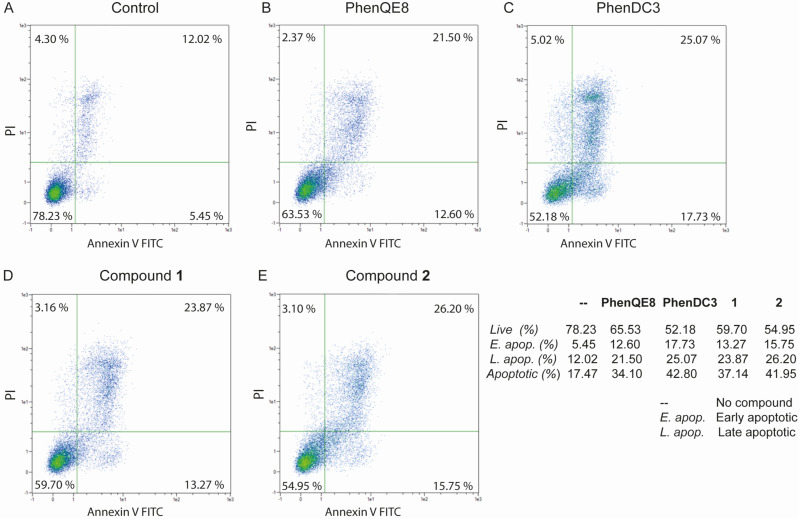
Annexin V/PI FITC apoptosis assays with HeLa cells treated with **1**, **2** or reference compounds PhenDC3 or PhenQE8, at 20 μM concentration for 48 h. Dot plots were obtained after flow cytometry analysis. Dot plot quadrants represent early apoptotic cells (**lower right**), late apoptotic cells (**upper right**), live cells (**lower left**) and necrotic cells (**upper left**).

Cell cycle analysis experiments were also carried out in combination with the Annexin V-PI FITC apoptosis assay, in HeLa cells. This time, cells were treated with compound **1**, compound **2** or antitumor controls for 48 h at a fixed, common concentration of 20 μM, and cell cycle effects and apoptosis induction were analyzed in parallel from the same batch of treated cells using flow cytometry. As shown in Figure 7 and Figure 8, compounds **1** and **2** were found to possess pro-apoptotic properties in HeLa cells at a 20 μM concentration.

Cell cycle experiments corroborated that compounds **1** and **2** cause an important increase in the number of cells in SubG0 (Figure 7D,E), while the Annexin V-PI assay was used to established whether apoptosis was involved in the cell death produced by the TSC derivatives. Dot plots also allowed us to distinguish percentage variations in the cell populations in early apoptosis from late-stage apoptosis, in comparison to untreated cells employed as controls (Figure 8). Compounds **1** and **2** were found to produce significant apoptosis after 48 h, with a notable increase (~20–25%) in the number of early apoptotic and late apoptotic cells (Figure 8D,E). As a matter of fact, the effect produced by compound **1** and **2**, especially the latter, is equivalent to the activity observed with antitumor compound PhenDC3 (Figure 8C). Interestingly, although compounds **1** and **2** were found to be less cytotoxic in MTT assays than analogue PhenQE8, their preliminary biological action by cell cycle and apoptosis assays in HeLa cells (20 μM, 48 h) is slightly superior (Figure 7B,D,E and Figure 8B,D,E). This highlights that the potential antitumor properties of the core structure of this family of TSC derivatives are maintained when the nitrogen atom of the guanidine group is replaced by a sulfur in a thiourea moiety.

## 4. Discussion

We carried out the synthesis of four 1,10-phenanthroline derivatives disubstituted at 2,9-positions as potential DNA ligands endowed with antitumor properties in vitro. Compounds **1**–**3** are thiosemicarbazone-based compounds (TSCs), either in neutral form or as ionic salts (PF_6_¯ and BF_4_¯, respectively), and compound **4** is a 4-phenylthiazole analogue, synthetically accessible from derivative **1**. To the best of our knowledge, we describe for the first time the DNA binding properties of known compound **1** and these three analogues, providing experimental results that support their potential applications as G-quadruplex DNA ligands and as cytotoxic agents with antitumor properties. Compared to our previous work on the closest structurally related analogue, the PhenQE8 G-Quadruplex ligand, the 1,10-phenanthroline heterocyclic core is substituted with two thiosemicarbazone units (TSCs), replacing two nitrogen atoms of the parent ligand for two sulfur atoms in compounds **1**–**3**. The synthesized derivatives are poly-nitrogenated compounds that lack additional aromatic or heteroaromatic rings, apart from the heterocyclic 1,10-phenantrholine system, rings that are normally present in other well-known G4 ligands and make an important contribution to maximize end stacking interactions with the quadruplex structure. Likewise, for PhenQE8 [29], X-ray diffraction studies [34] indicate that these molecules can adopt extended conformations, and this may be the reason why they are found to be optimal for quadruplex recognition, besides the fact that they possess only one heteroaromatic ring in their structure.

In this work, the human telomeric sequence Tel22 was chosen as the target G4 structure, as it constitutes one of the most representative examples of biologically relevant DNA quadruplex. In particular, we focused on the two hybrid structures, which are present in solution when the quadruplex is folded in K^+^ ionic conditions. The study of the in vitro interactions of compounds **1**–**4** with the telomeric quadruplex was approached by using several techniques, such as FRET-based DNA melting assays, CD and competitive and non-competitive equilibrium dialyses. Furthermore, the binding mode of compound **1** with CT DNA was investigated through viscometric titrations.

These DNA interaction studies show that compounds **1**–**3** can thermally stabilize the telomeric quadruplex DNA to a greater extent than compound **4**, the 4-phenythiazole derivative, whereas they produce negligible stabilization effects on double-stranded DNA. The degree of stabilization of the telomeric structure was very similar in the three compounds, although **2** exhibited slightly higher ΔTm values. Compound **1**, representing the core TSC structure of the family, was selected and studied in further detail. Circular dichroism spectra revealed that compound **1** stabilizes the hybrid structure of the telomeric G4 DNA, at least up to a ratio 1:5 Tel22/ligand and, most importantly, it induces a conformational change similar to that produced by ligand PhenQE8, increasing the antiparallel character of the structure. However, unlike PhenQE8, an apparent ICD band was observed at 350 nm for the 1:5 Tel22/ligand ratio, where the compound absorbs, suggesting that the derivative, besides end-stacking interactions with the telomeric quadruplex, may have other additional binding sites as a groove binder, or even as a partial intercalating agent.

We determined through equilibrium dialysis experiments that compound **1** interacts with quadruplex and dsDNA with different binding affinities, with an apparent association constant to the telomeric quadruplex of ~10^5^ M^−1^, while its binding affinity to duplex DNA is significantly lower (~5 × 10^3^ M^−1^). Under competitive conditions, the selectivity of **1** to preferentially bind the telomeric quadruplex Tel22 over ds17 dsDNA was estimated to be ~24-fold. This represents a notable decrease in quadruplex versus dsDNA selectivity, compared to ligand PhenQE8. Overall, the replacement of two nitrogen atoms in PhenQE8 by two sulfur atoms seems to produce a profound effect on DNA binding affinity, although the derivatives still retain a certain degree of selectivity for the quadruplex structure. In addition, viscosity titration assays with CT DNA demonstrated that compound **1** does not behave as a DNA intercalating agent, but it recognizes the double helix by groove or electrostatic external binding, similar to PhenQE8. Future molecular modeling studies, including docking and molecular dynamics simulations, will be used to shed light on the binding mechanism of these derivatives with the telomeric and other G4 DNA sequences.

The in vitro antitumor properties of compounds **1**–**4** were preliminarily assessed in cultured cells by using the MTT viability assay, cell cycle analysis and the annexin V-PI apoptosis assay. The TSCs compounds (**1**–**3**) showed modest cytotoxic activity in the cell lines selected for this study (PC-3, DU-145, HeLa, MCF-7 and HT-29) with micromolar IC_50_ values, whereas compound **4** did not prove active in any of the cell lines tested. Overall, compounds **1**–**3** exhibited a lower cytotoxicity than the G4 ligand PhenQE8 and cisplatin metallodrug in all cell lines tested but a similar or higher cytotoxicity than PhenDC3 in the case of PC3 and HeLa cells. The decreased cytotoxicity with respect to PhenQE8 is more pronounced for the neutral derivative which, in contrast, was the only compound that displayed a modest degree of selectivity in prostate cells (prostate tumor PC3 versus prostate non-tumor RWPE-1, selectivity index (SI)~5). In general, within the TSC compounds, those in the form of ionic salts were found to be more cytotoxic to tumor and normal cells than the neutral derivative.

Finally, compounds **1** and **2** have been shown to alter cell cycles in HeLa cells after 48 h of treatment, with a considerable increase in the number of cells in the SubG0 phase, which suggests that the compounds can produce cell death by apoptosis. This was further confirmed through the Annexin-PI apoptosis assay, which also revealed a significant increase in the number of cells in early and late apoptosis stages, which was more pronounced in the case of compound **2**. The observed effect is similar to that produced by antitumor compound PhenDC3 and slightly superior to the activity observed for analogue PhenQE8. A further investigation on the mechanism of action of this family of compounds will be needed to precisely determine the molecular targets of PhenQE8 and TSC analogues that account for their biological activity.

## 5. Conclusions

In this work, a series of four 2,9-disubstituted 1,10-phenanthroline derivatives were synthesized, three of them representing novel compounds. The compounds represent thiosemicarbazones (TSCs), neutral compound **1,** and two ionic derivatives (PF_6_¯ and BF_4_¯ salts, **2** and **3**, respectively), with the fourth being a 4-phenylthiazole derivative (**4**). Their binding interactions with telomeric quadruplex and with dsDNA, especially for derivative **1**, were assessed using several techniques, from which we conclude that the TSC derivatives retain the ability to bind the telomeric quadruplex with more binding affinity than with dsDNA, as other known 1,10-phenanthroline-based 2,9-disubstituted quadruplex ligands, although they demonstrated only a moderate selectivity. Compounds **1**–**3** proved cytotoxic against various tumor cell lines (PC-3, DU145, HeLa, MCF-7 and HT29) and against the two normal cell lines tested (HFF-1 and RWPE-1), with only compound **1** showing minor selectivity in prostate cells (SI = 5). On the other hand, the modification of the structure with the incorporation of a thioazole ring in the side chain of these 2,9-substituted heterocyclic systems clearly diminishes its thermal stabilization of the quadruplex DNA structure, and, most importantly, it abolishes cytotoxic activity below a 100 μM concentration. Although the cytotoxicity of derivatives **1**–**3** in tumor cells is moderate compared to other well-known G4 ligands, the compounds are able to produce cell death by apoptosis, an effect that compares well to, for example, PhenDC3 or PhenQE8 biological activities.

The poly-nitrogenated nature of the reported compounds and the extended conformations that these molecules are known to adopt may account for their capacity to recognize and bind G4 DNA structures with superior affinities than their double-stranded counterparts, despite the lack of additional aromatic rings besides the 1,10-phenanthroline system. However, this work brings to the fore that the replacement of two nitrogen atoms in the related PhenQE8 analogue by two sulfurs in the TSCs compounds results in a decrease in overall binding affinity for DNA secondary structures and a diminished cytotoxicity and selectivity, although it preserves the ability to induce apoptosis in tumor cell lines. Further investigations will be required to assess the scope and applications of the reported derivatives as potential antitumor agents.

## Figures and Tables

**Table 1:** Binding constants (K_app_) of **1** with G4 Tel22 and with ds17 DNA determined by no-competition and competition equilibrium dialyses (10 mM potassium phosphate buffer, 100 mM KCl, pH = 7.2).

	1 + Tel22	1 + ds17	1 + Tel22 + ds17
DNA	K_app_ (M^−1^) × 10^−5^	K_app_ (M^−1^) × 10^−5^	K_app_ (M^−1^) × 10^−5^
Tel22 (Q)	1.1 ± 0.1	--	1.2 ± 0.1
ds17 (D)	--	0.06 ± 0.01	0.05 ± 0.01

**Table 2:** IC_50_ values of derivatives **1**–**4** and selected known antitumor compounds (PhenQE8, PhenDC3 and cisplatin) determined through the MTT viability assay after a 72 h treatment using various tumor and normal cell lines: PC-3, DU145 (prostate), HeLa (cervix), MCF-7 (breast), HT29 (colorectal), HFF-1 (healthy fibroblasts) and RWPE-1 (healthy prostate). N.D.: Not determined.

Ligand	PC-3	DU145	HeLa	MCF-7	HT29	HFF-1	RWPE-1
	IC_50_ (μM)	IC_50_ (μM)	IC_50_ (μM)	IC_50_ (μM)	IC_50_ (μM)	IC_50_ (μM)	IC_50_ (μM)
1	18.4 ± 4.9	62.2 ± 5.9	~100	78.4 ± 11.0	> 100	74.4 ± 3.1	90.8 ± 8.8
2	41.6 ± 2.8	82.5 ± 3.0	71.2 ± 4.6	39.7 ± 4.6	48.5 ± 6.7	72.9 ± 8.8	11.5 ± 1.3
3	33.8 ± 4.7	48.6 ± 1.4	42.6 ± 7.1	39.6 ± 4.8	49.7 ± 3.7	69.1 ± 5.0	17.3 ± 0.7
4	>>100	>>100	>>100	>>100	>>100	>>100	>>100
PhenQE8	5.6 ± 0.3	22.7 ± 1.1	16.2 ± 1.6	44.9 ± 9.2	N.D.	170.0 ± 23.3	38.8 ± 0.5
PhenDC3	55.5 ± 3.1	7.3 ± 1.0	>100	2.4 ± 0.8	N.D.	32.2 ± 4.2	8.2 ± 1.1
cisplatin	12.8 ± 0.9	8.4 ± 1.5	18.0 ± 1.9	96.5 ± 6.7	86.6 ± 7.0	36.6 ± 4.4	16.4 ± 1.4

## Data Availability

Data are contained within the article and supplementary materials.

## Supplementary Materials

The following supporting information can be downloaded at: https://www.mdpi.com/article/10.3390/biology13010060/s1, including NMR and HRMS spectra of compounds **1**–**4** (Figures S1–S14 and S15–S18, respectively) and cell cycle histograms (Figure S19).
