# Supplementary material for: DNA Interactions and Biological Activity of 2,9-Disubstituted 1,10-Phenanthroline Thiosemicarbazone-Based Ligands and a 4-Phenylthiazole Derivative
Source: Biology (Basel). 2024 Jan 20;13(1):60. doi: 10.3390/biology13010060 (PMC10813753; doi:10.3390/biology13010060)
Supplement: Supplementary file 1 [file biology-13-00060-s001.zip › biology-2823733-supplementary.pdf]

DNA interactions and biological activity of 2,9-disubstituted 1,10-phenanthroline thiosemicarbazone-based ligands and a 4-phenylthiazole derivative

Álvaro Nicolás,[a,b] Julia G. Quero,[a] Marta Barroso,[a] Zoila Gándara,[a,b*] and Lourdes Gude[a,b*]

[a]*Universidad de Alcalá, Departamento de Química Orgánica y Química Inorgánica, Instituto de Investigación Química "Andrés M. del Río" (IQAR), 28805-Alcalá de Henares, Madrid, Spain.*
[b]*Grupo DISCOBAC, Instituto de Investigación Sanitaria de Castilla-La Mancha (IDISCAM), Spain.*

^{1}H, ^{13}C, ^{1}H-^{1}H-COSY, ^{1}H-^{13}C-HSQC and ^{1}H-^{13}C-HMBC NMR spectra of **1**........ Figures S1-S5

^{1}H, ^{13}C, ^{19}F and ^{31}P NMR spectra of **2** .... Figures S6-S9

^{1}H, ^{13}C and ^{19}F spectra of **3**........ Figures S10-S12

^{1}H and ^{13}C NMR spectra of **4**........ Figures S13, S14

Zoomed view of high-resolution electrospray ionization mass spectrum (ESI-HRMS-pos) of **1** (MeOH, 1% formic acid) .... Figure S15

Zoomed view of high-resolution electrospray ionization mass spectrum (ESI-HRMS-pos) of **2** (MeOH, 1% formic acid)........ Figure S16

High-resolution electrospray ionization mass spectrum (ESI-HRMS-pos) of **3** (MeOH, 1% formic acid)........ Figure S17

Zoomed view of high-resolution electrospray ionization mass spectrum (ESI-HRMS-pos) of **4** (MeOH, 1% formic acid)........ Figure S18

Cell cycle assay histograms obtained by flow cytometry in HeLa cells treated with **1** or **2** (1/2 IC_{50} concentrations, 72 h)........ Figure S19

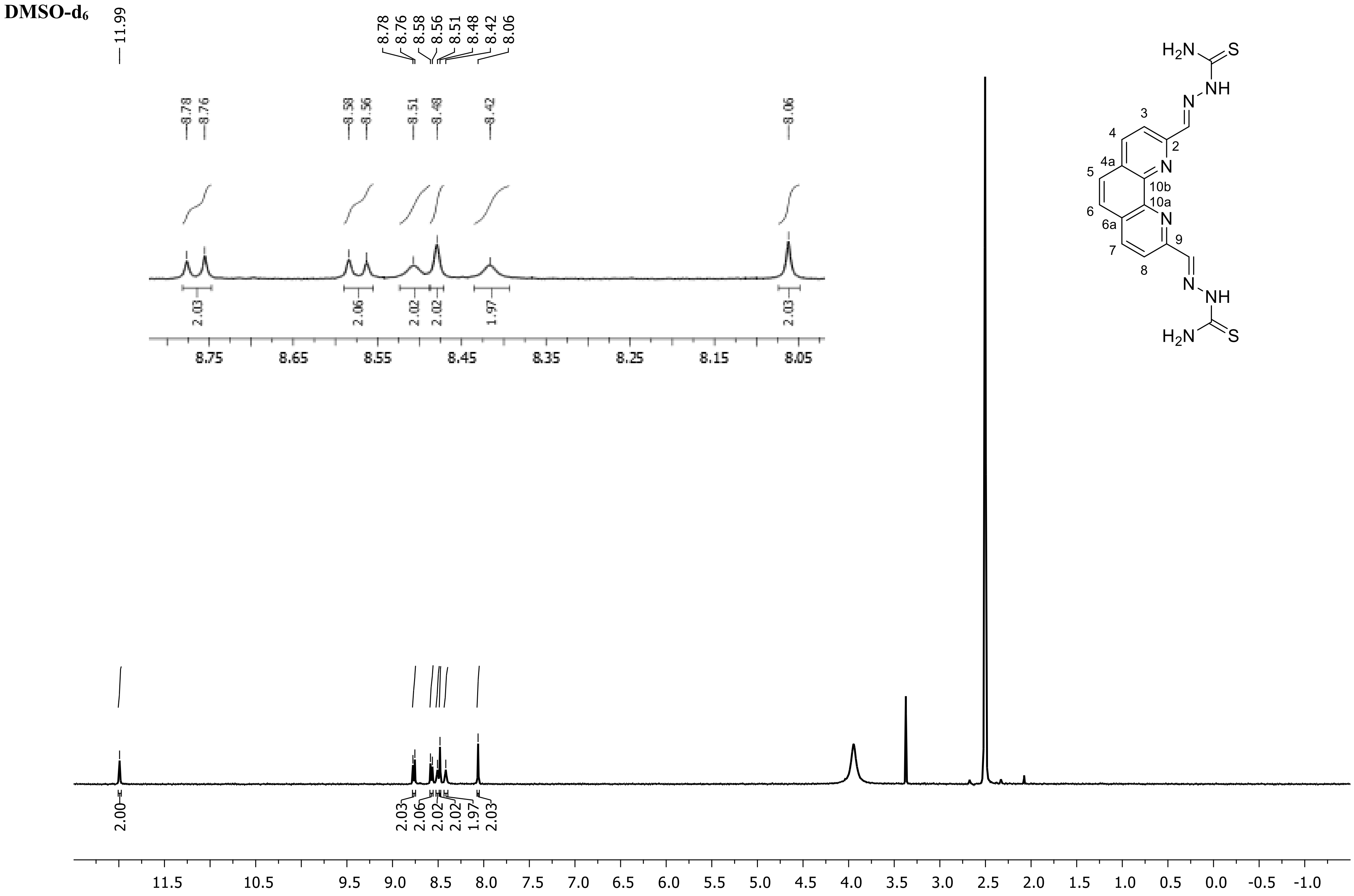

Figure S1. ^{1}H NMR (400 MHz, DMSO-d_6) of **1**

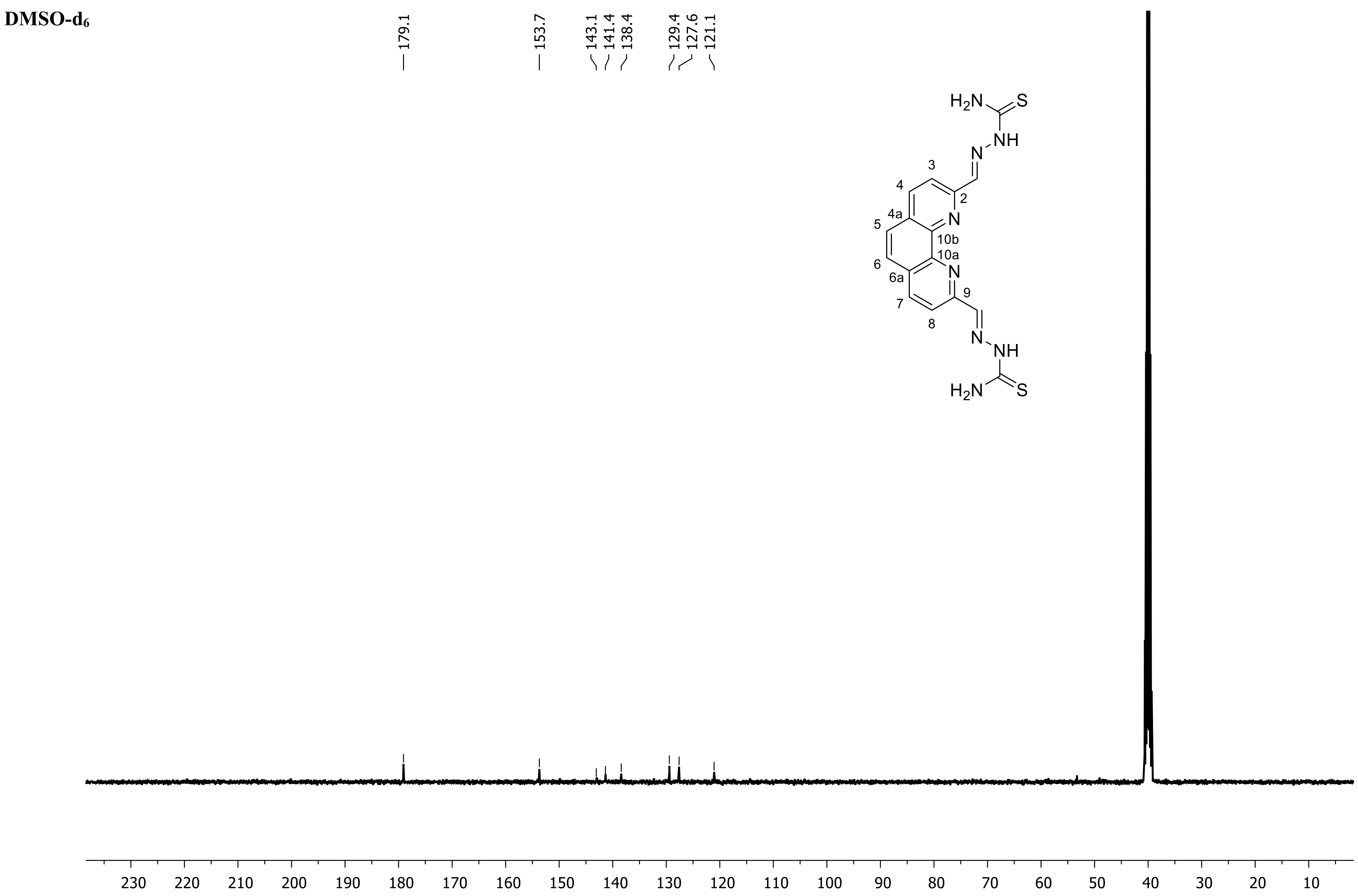

Figure S2. ^{13}C NMR (101 MHz, DMSO-d6) of **1**

DMSO-d$_6$

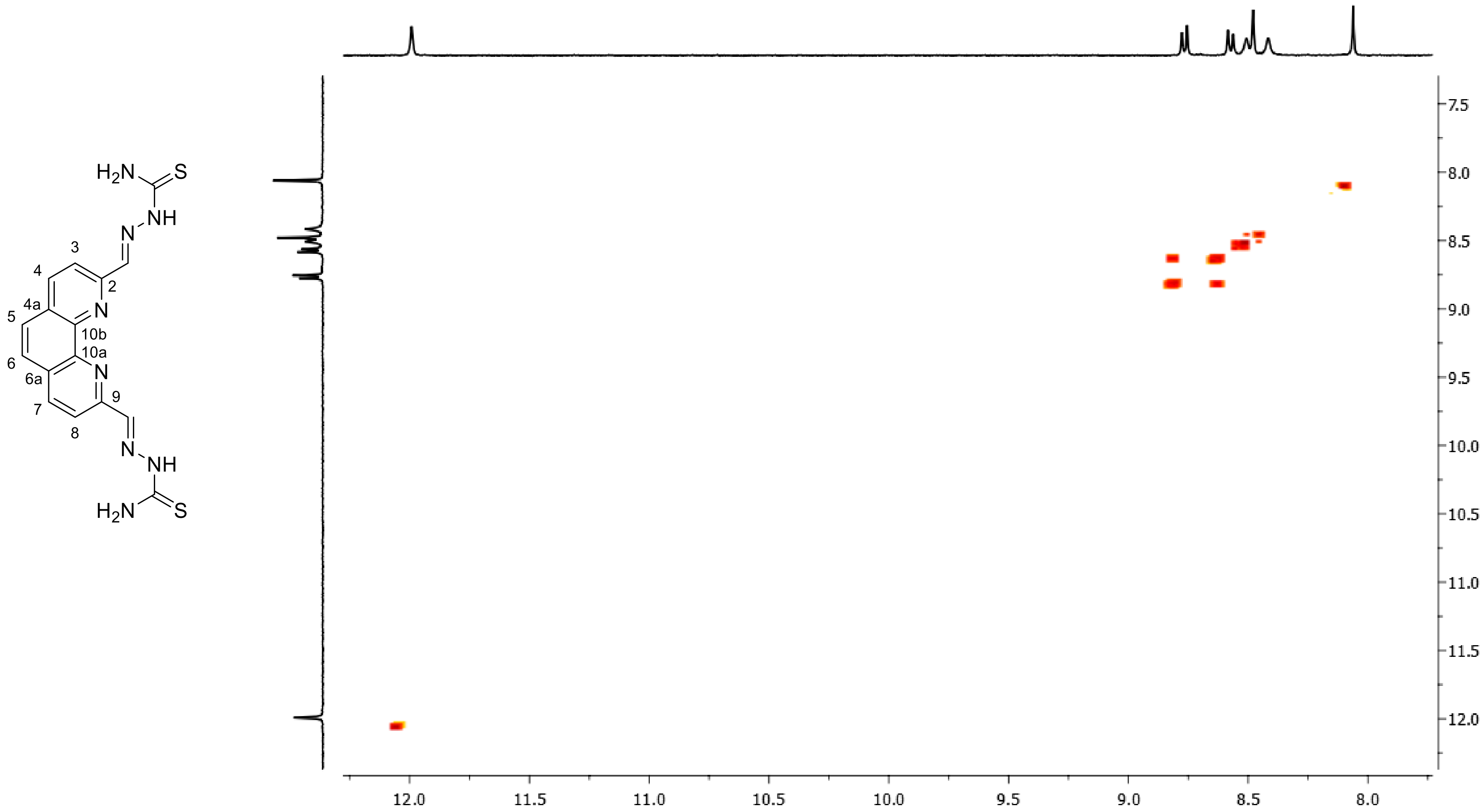

Figure S3. ^{1}H-^{1}H-COSY (400 MHz, DMSO-d6) of **1**

DMSO-d$_6$

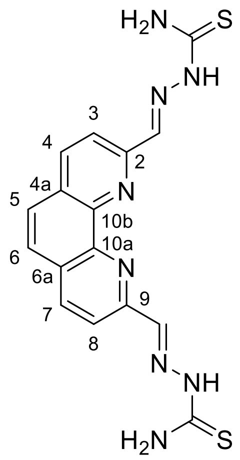

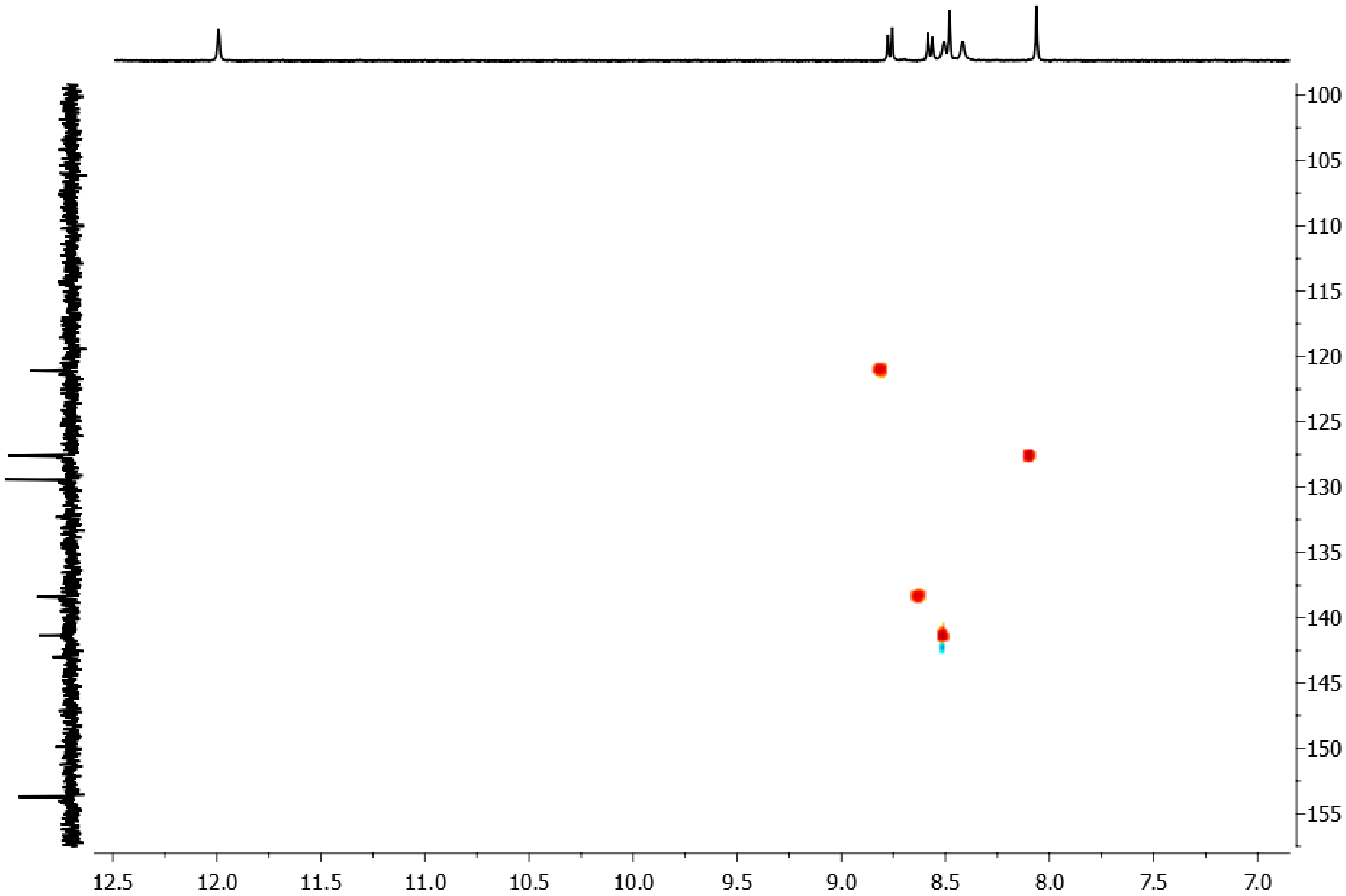

Figure S4. ^{1}H-^{13}C-HSQC (400 MHz, DMSO-d6) of **1**

DMSO-d₆

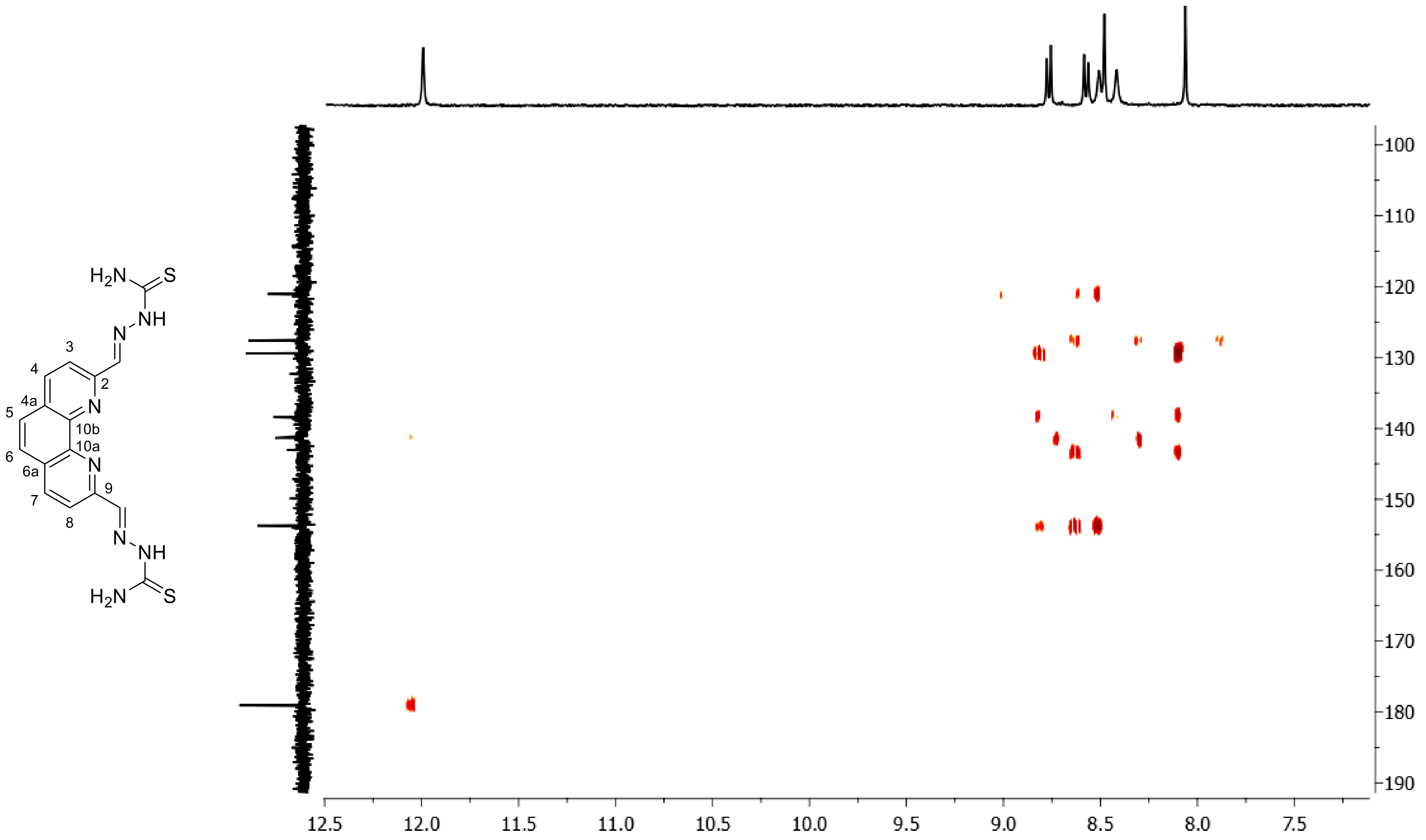

Figure S5. ^{1}H-^{13}C-HMBC (400 MHz, DMSO-d6) of **1**

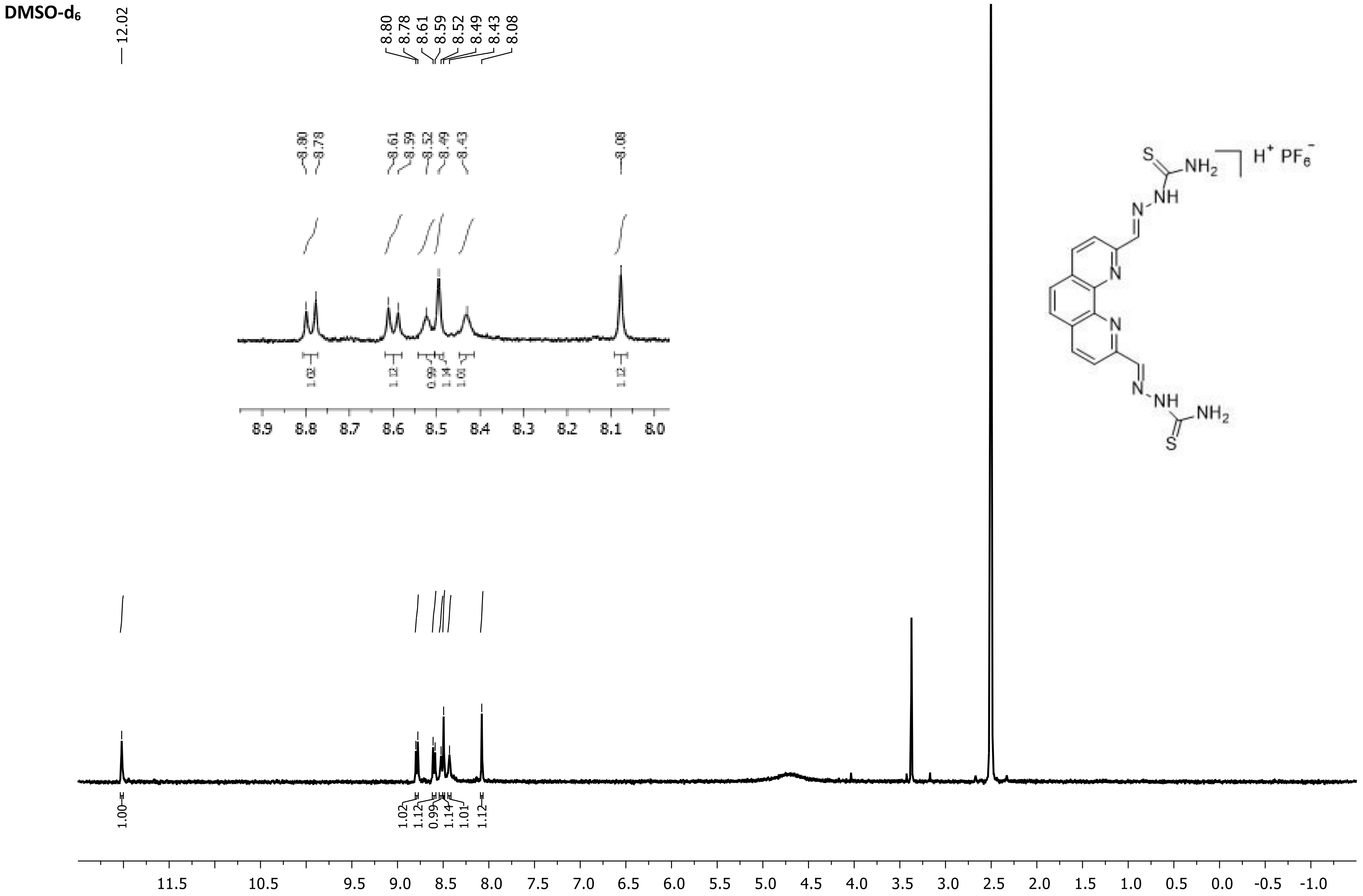

Figure S6. ^{1}H NMR (400 MHz, DMSO-d$_6$) of **2**

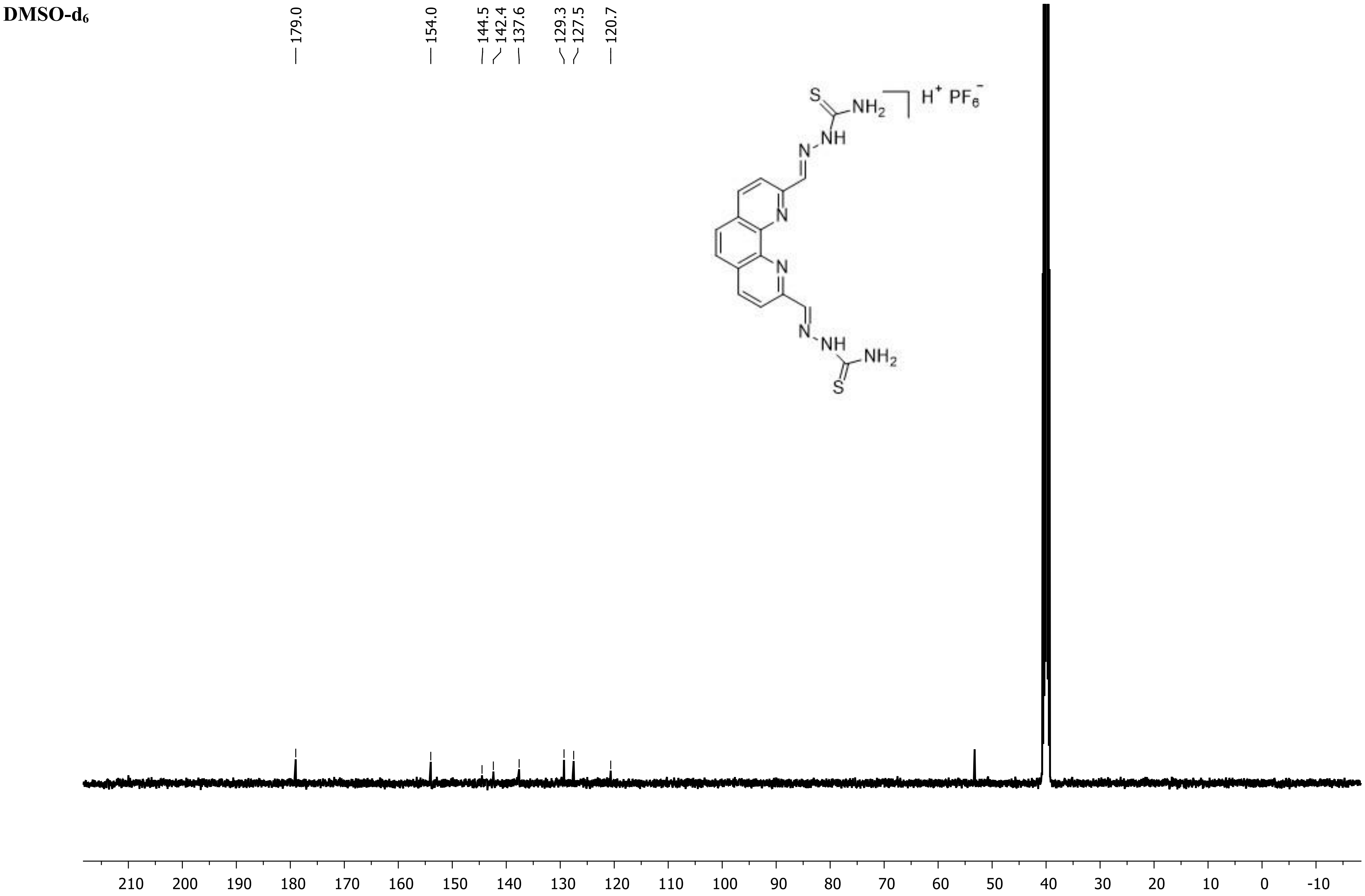

Figure S7. ^{13}C NMR (101 MHz, DMSO-d_6) of **2**

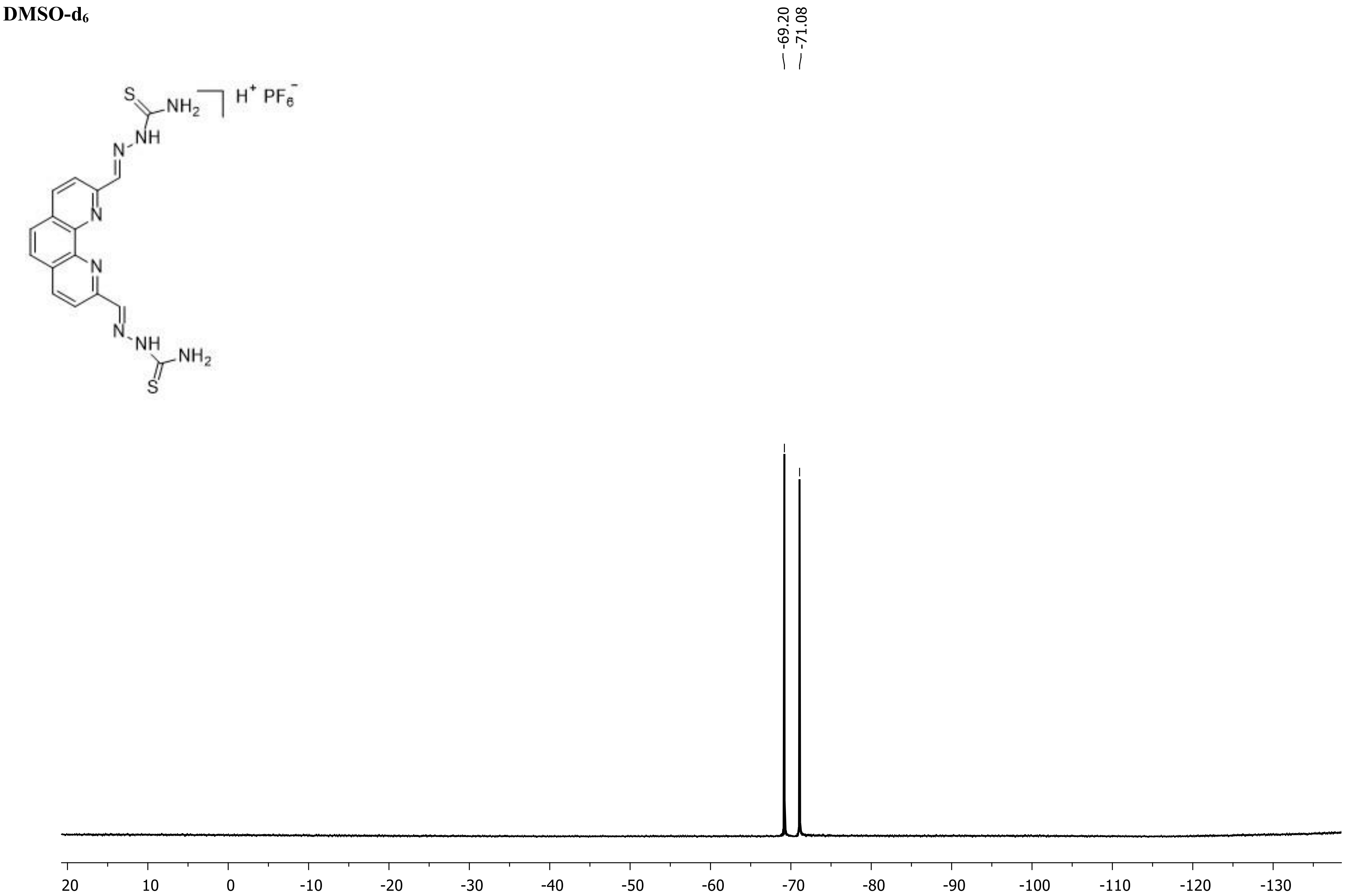

Figure S8. ^{19}F NMR (376 MHz, DMSO-d$_6$) of **2**

DMSO-d₆

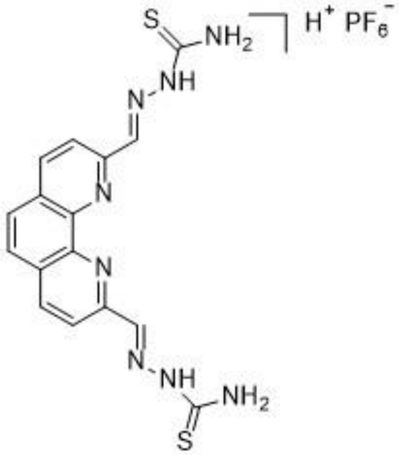

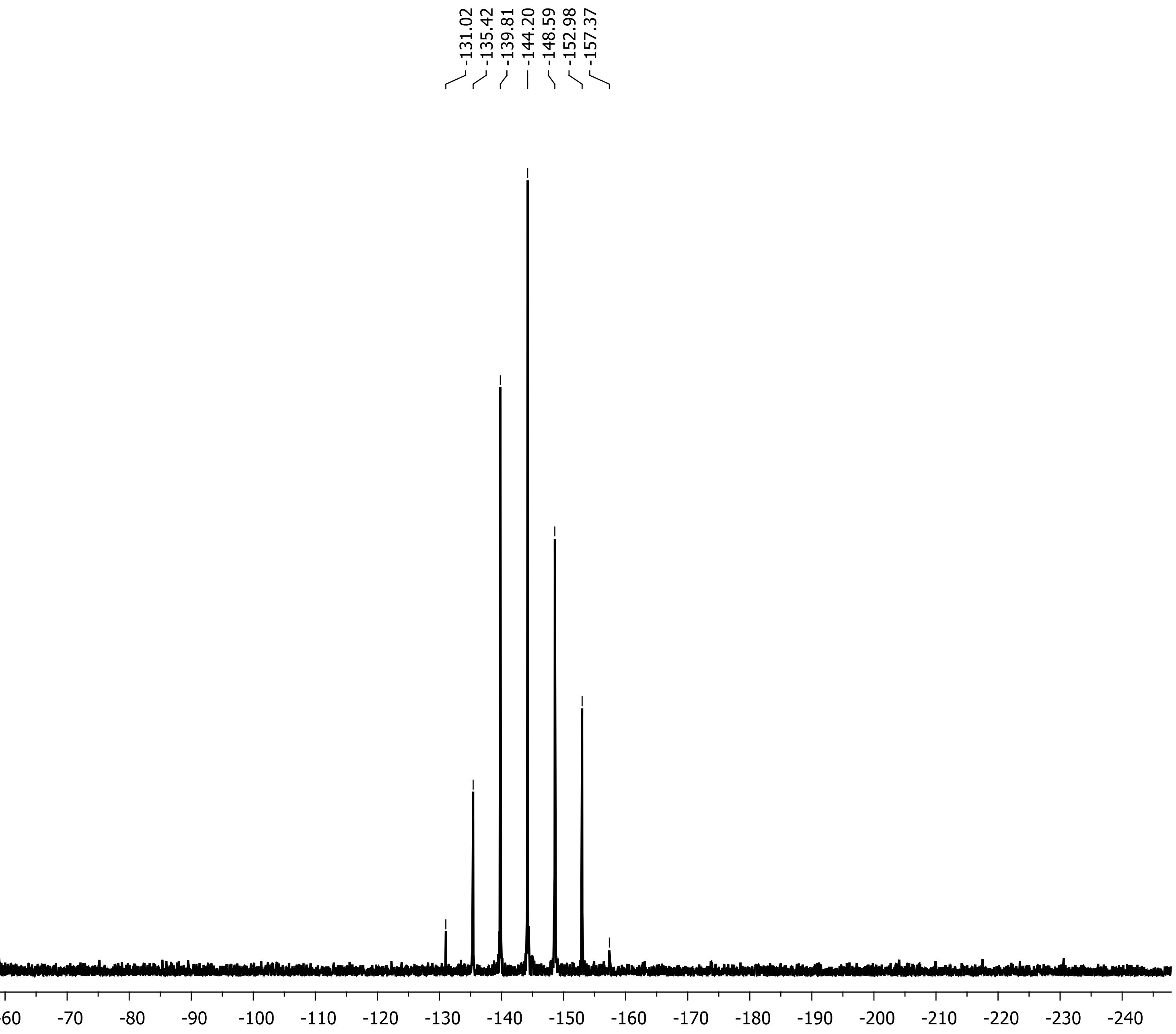

Figure S9. ^{31}P NMR (162 MHz, DMSO-d_6) of **2**

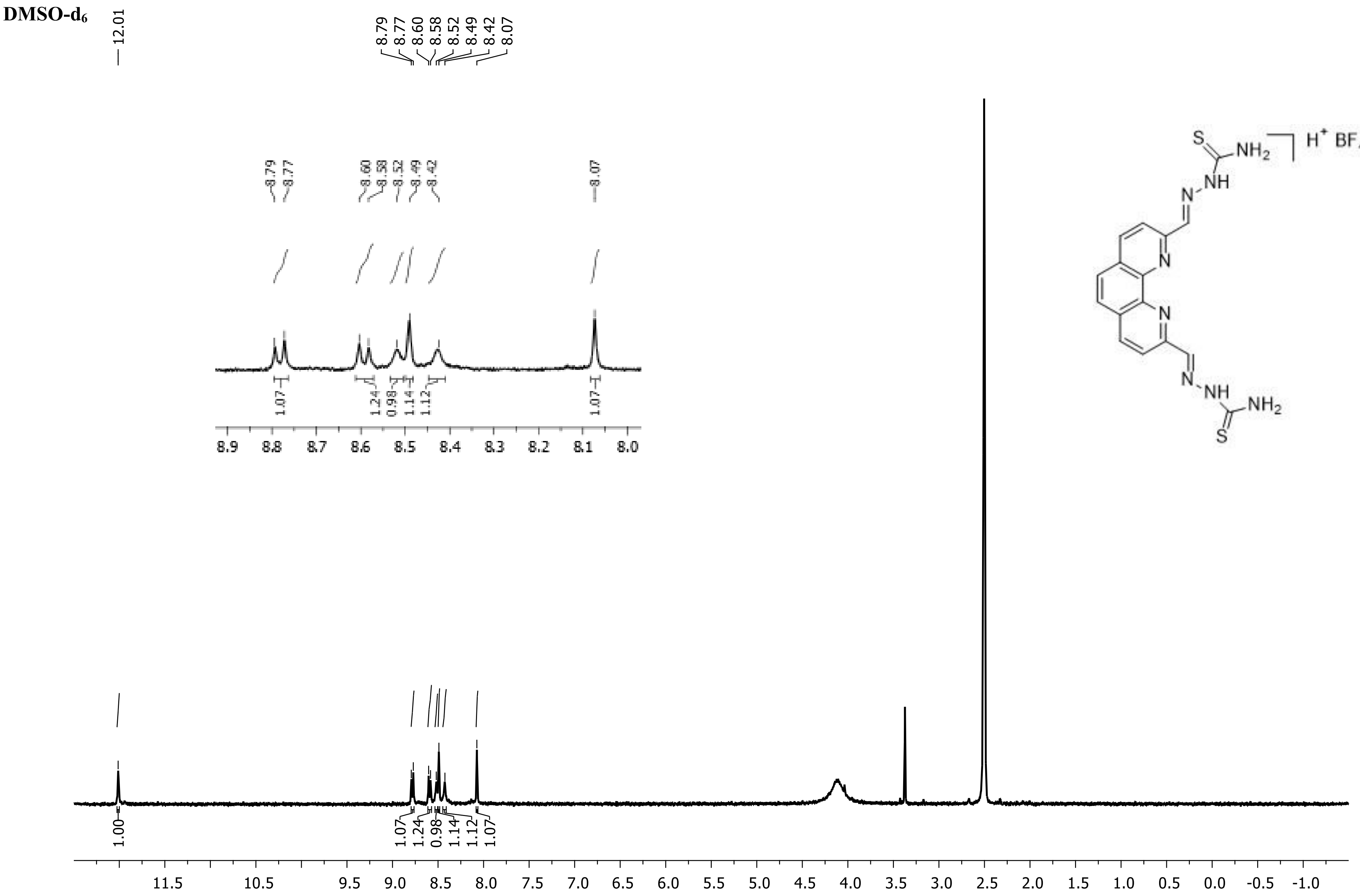

Figure S10. ^{1}H NMR (400 MHz, DMSO-d_6) of **3**

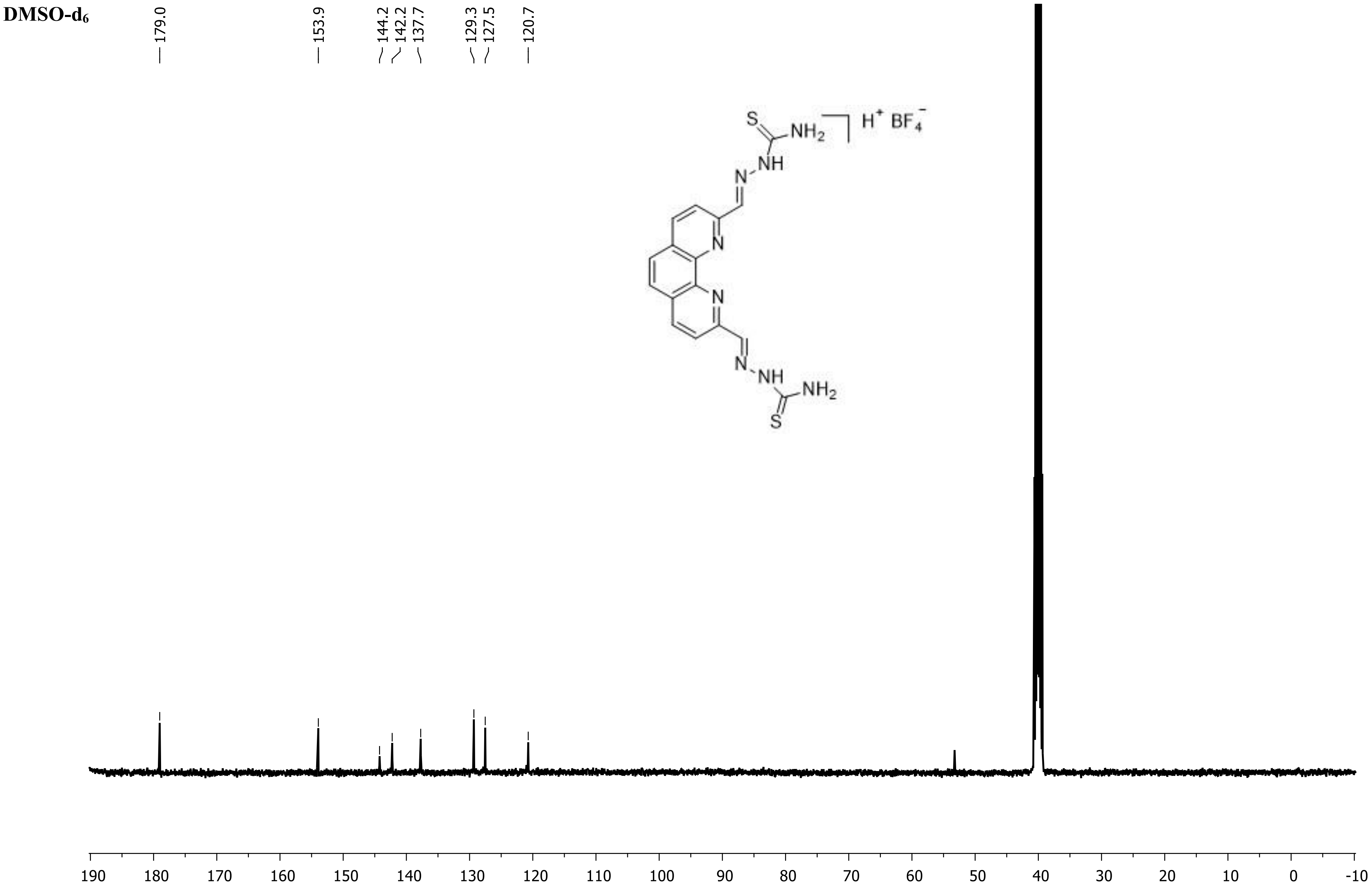

Figure S11. ^{13}C NMR (101 MHz, DMSO-d_6) of **3**

DMSO-d6

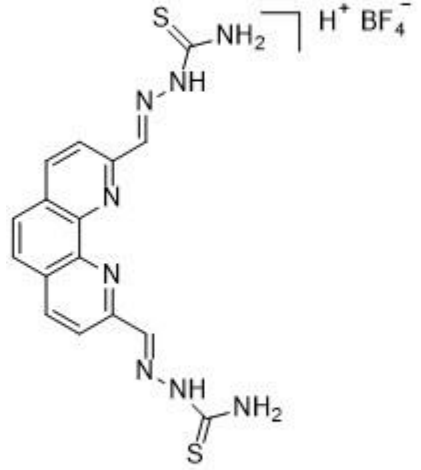

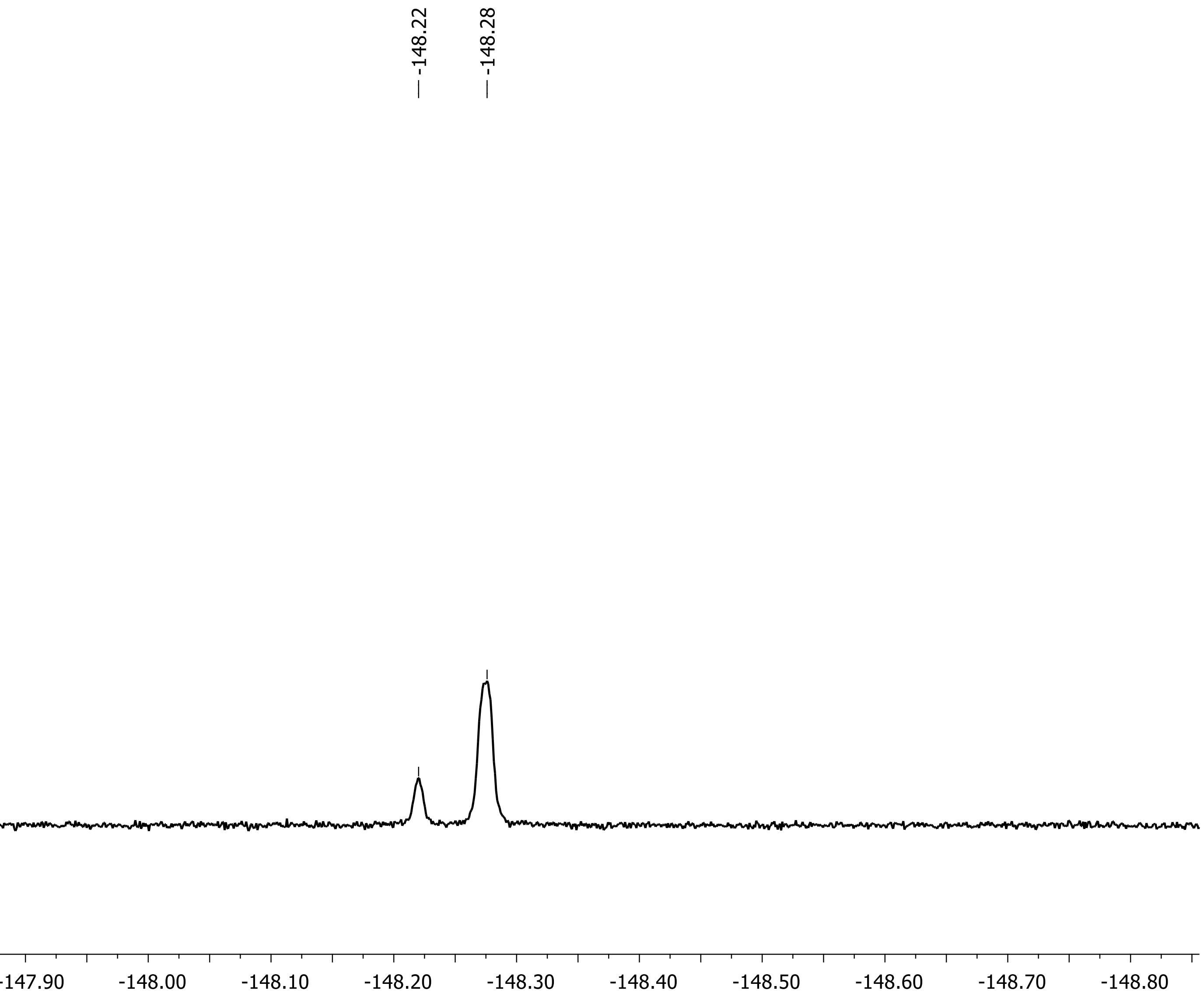

Figure S12. ^{19}F NMR ((376 MHz, DMSO-d$_6$) of **3**

DMSO-d$_6$

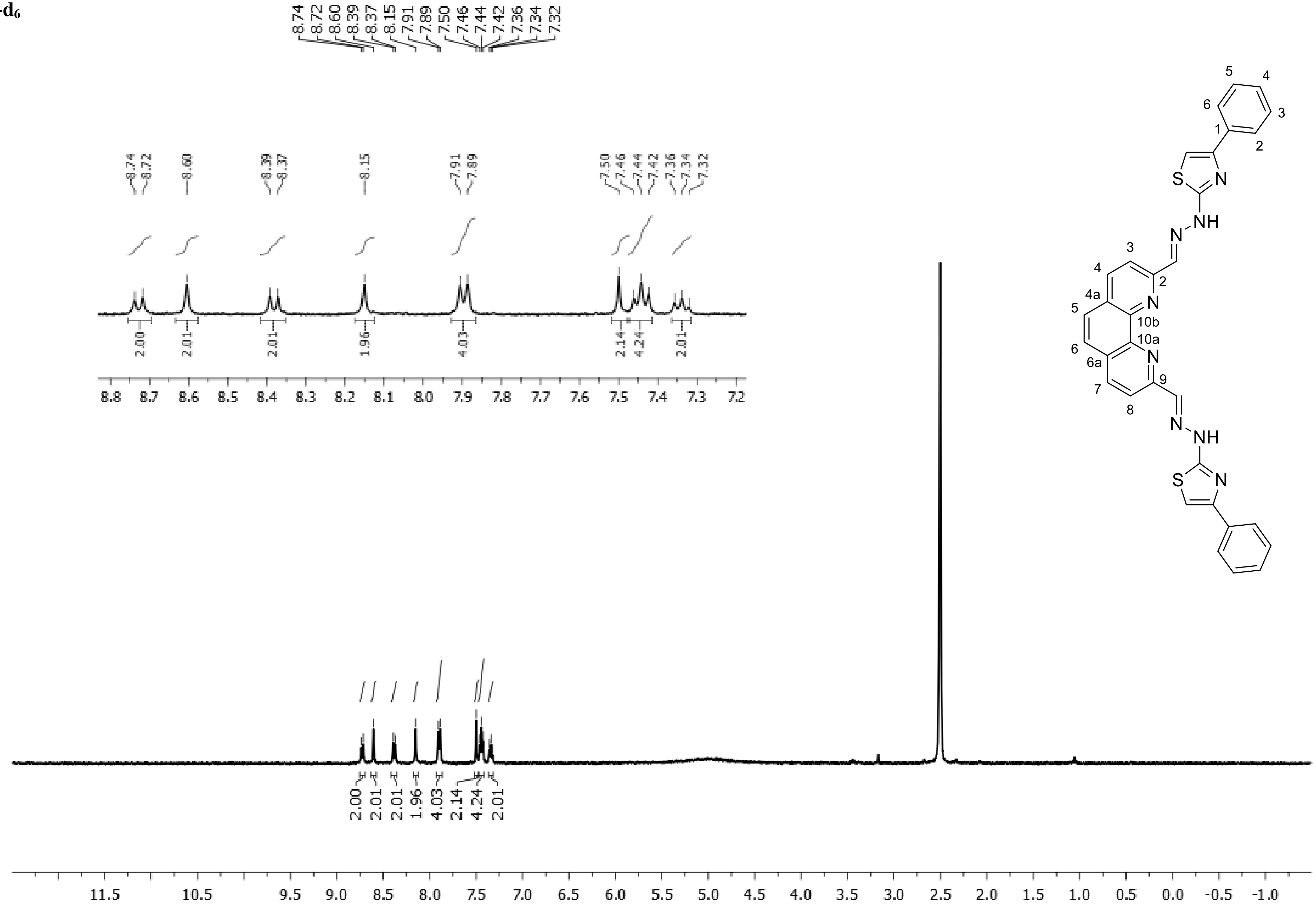

Figure S13. ^{1}H NMR (400 MHz, DMSO-d$_6$) of **4**

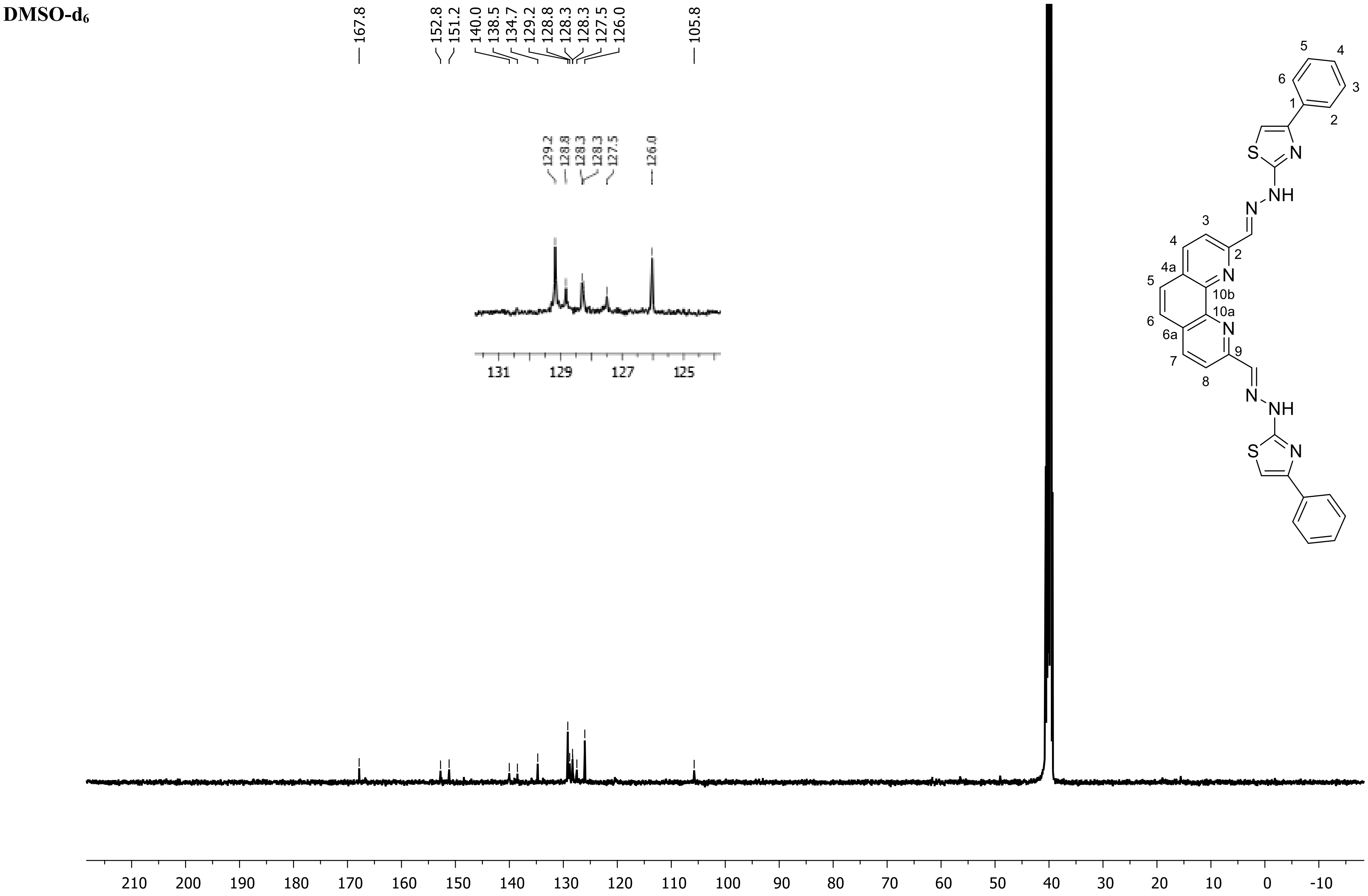

Figure S14. ^{13}C NMR (101 MHz, DMSO-d$_6$) of **4**

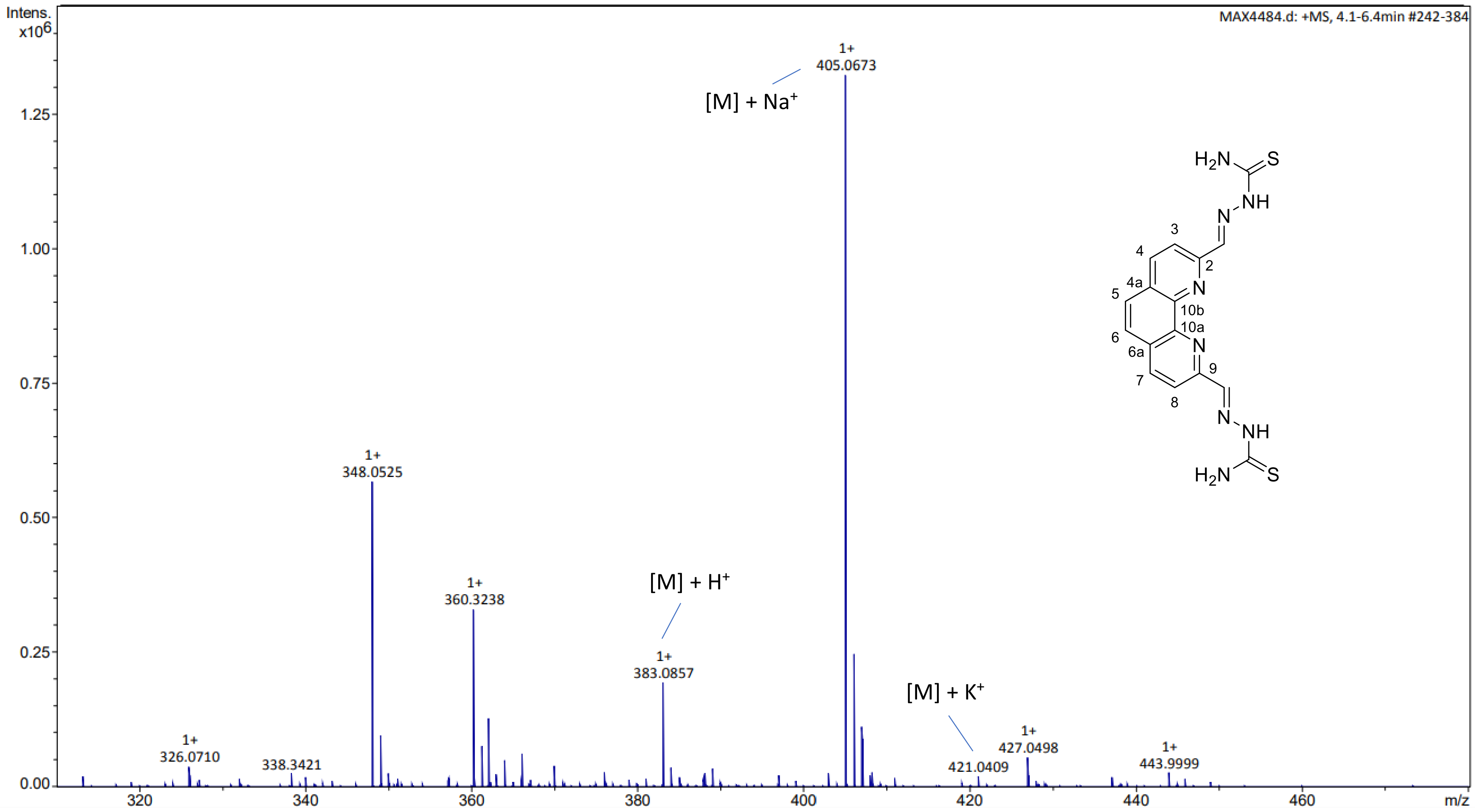

Figure S15. Zoomed view of high-resolution electrospray ionization mass spectrum (ESI-HRMS-pos) of **1** (MeOH, 1% formic acid)

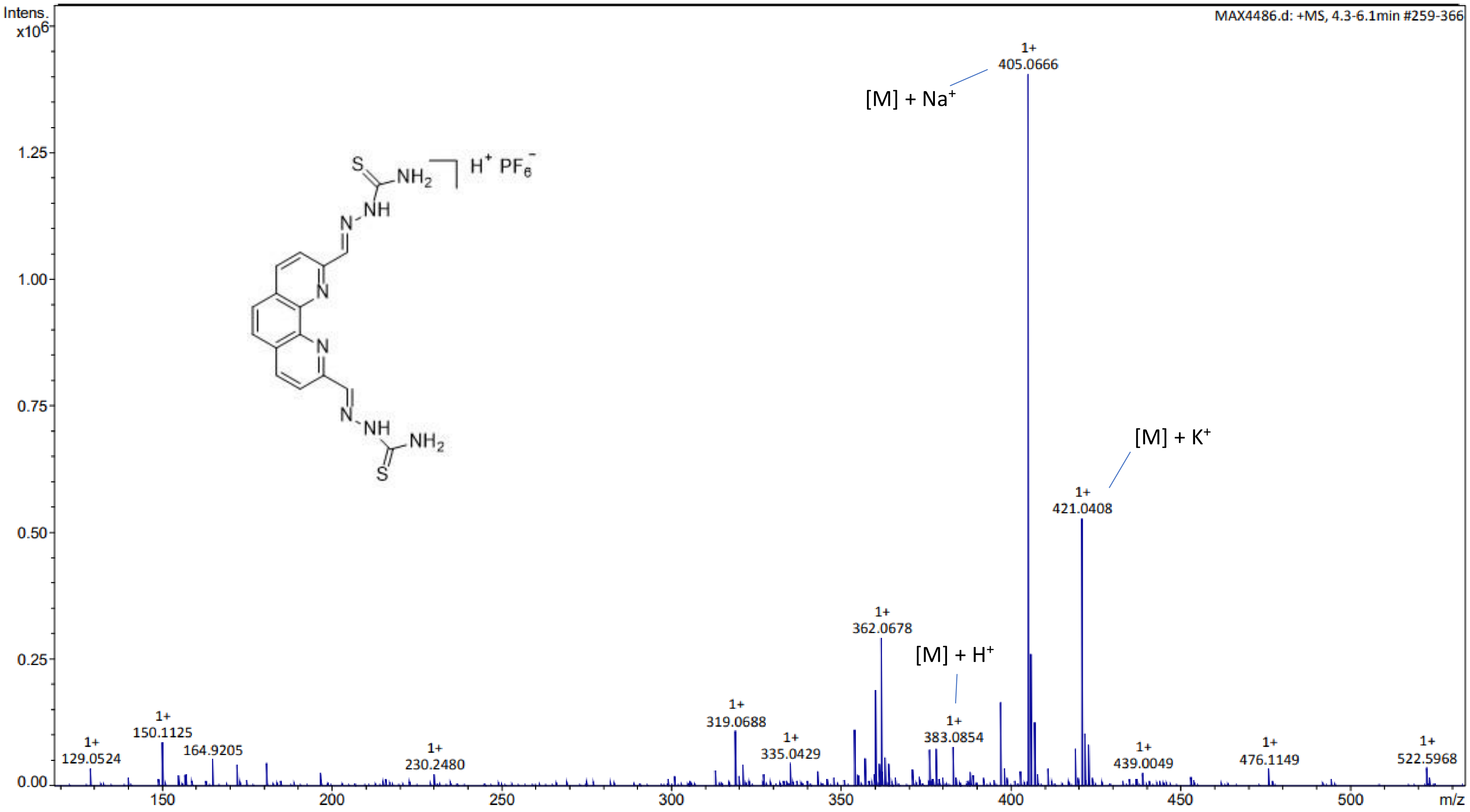

Figure S16. Zoomed view of high-resolution electrospray ionization mass spectrum (ESI-HRMS-pos) of **2** (MeOH, 1% formic acic)

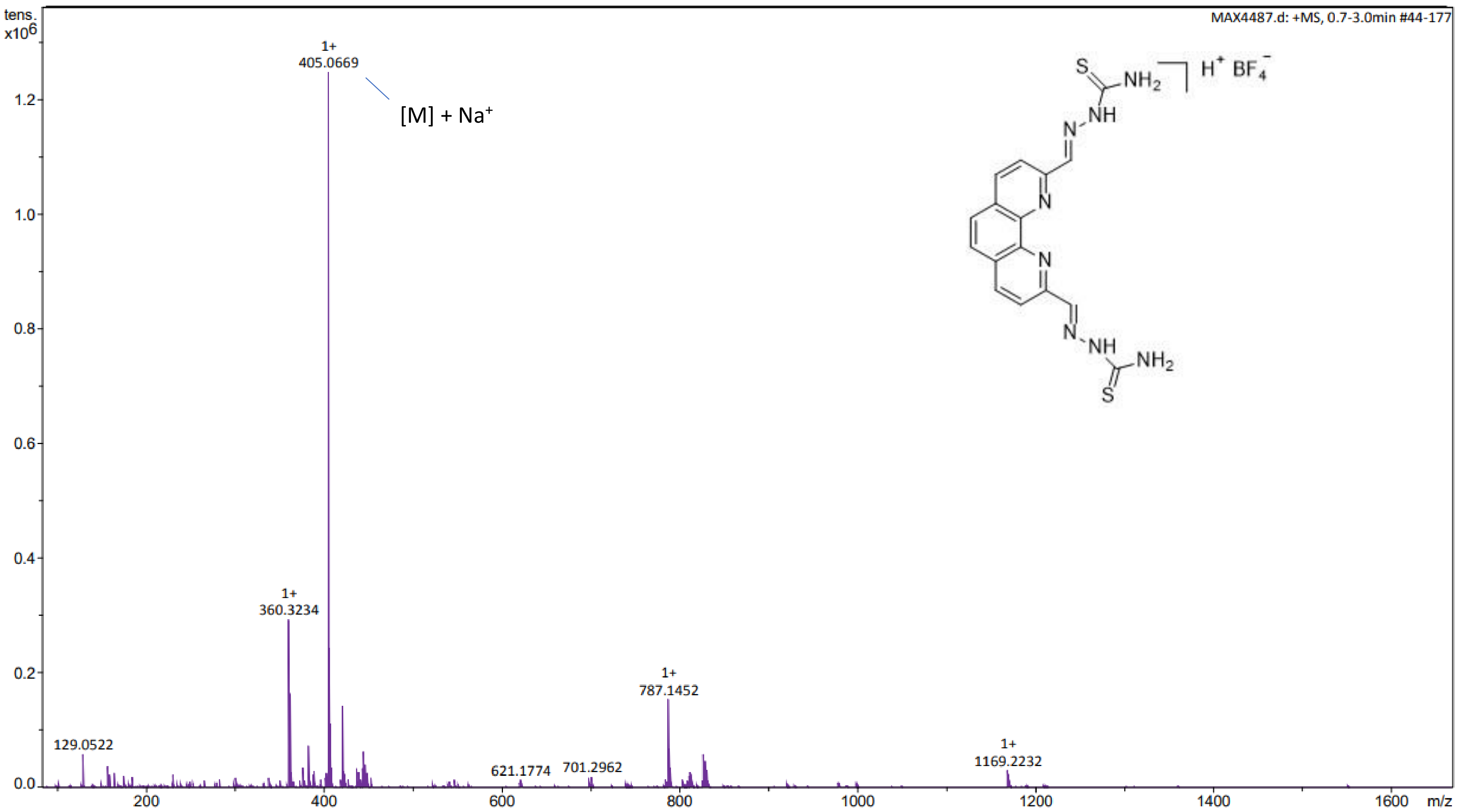

Figure S17. High-resolution electrospray ionization mass spectrum (ESI-HRMS-pos) of **3** (MeOH, 1% formic acid)

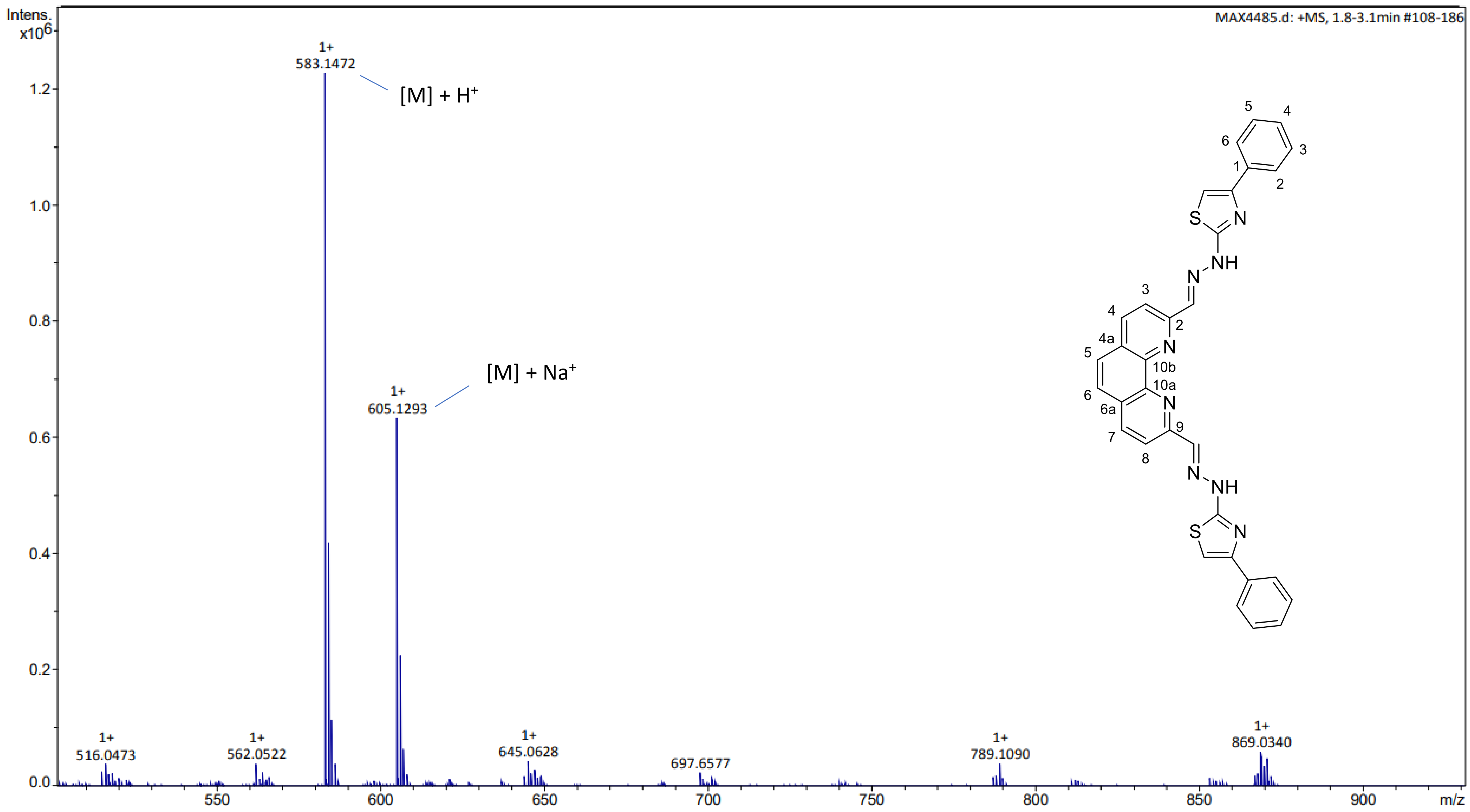

Figure S18. Zoomed view of high-resolution electrospray ionization mass spectrum (ESI-HRMS-pos) of **4** (MeOH, 1% formic acid)

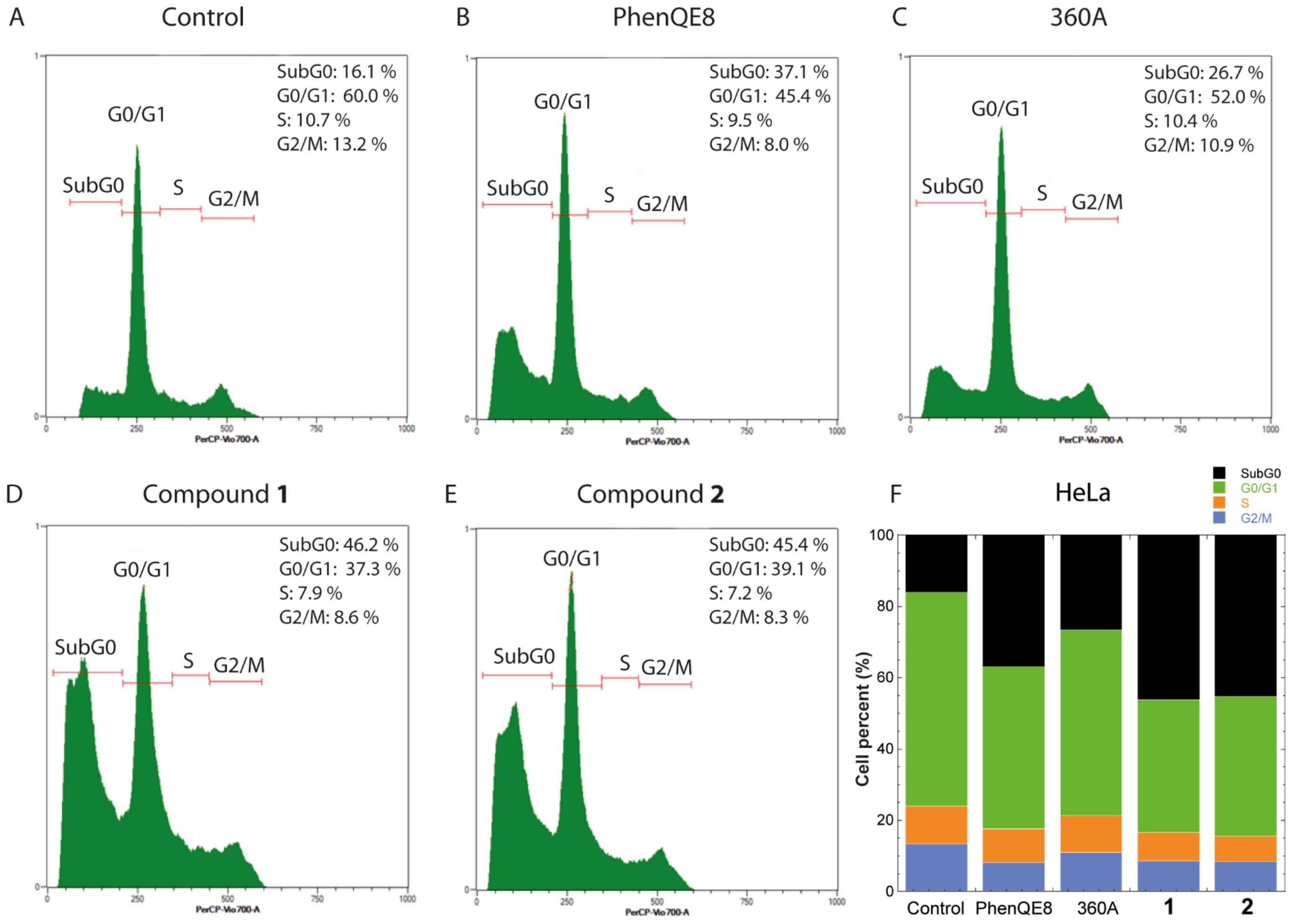

Figure S19. HeLa cell cycle histograms after 72 h treatment with ½ IC_{50} compound concentration. A) Untreated cells (negative control); B) PhenQE8 (structural analogue with antitumor properties, positive control); C) 360A (antitumor agent, positive control); D) compound **1**; E) compound **2**, and F) Stack bar graph representing averaged cell percents in each cycle phase and SubG0 after compound treatment. Increase in the SubG0 population percent is associated to apoptosis.
